# Influence of internal migration on antenatal care utilization in Bangladesh: Findings from a nationally representative cross-sectional survey

**DOI:** 10.1371/journal.pone.0345817

**Published:** 2026-06-26

**Authors:** Md. Minhajul, Md. Mahfuzur Rahman, Md Ismail Tareque

**Affiliations:** 1 Department of Population Science and Human Resource Development, University of Rajshahi, Rajshahi, Bangladesh; 2 Department of Geography, College of Arts & Social Sciences, Sultan Qaboos University, Muscat, Sultanate of Oman; Academic Medical Center: Amsterdam UMC Locatie AMC, NETHERLANDS, KINGDOM OF THE

## Abstract

Internal migration plays a crucial role in shaping healthcare utilization patterns, particularly in developing countries. However, its influence on maternal healthcare utilization, especially on antenatal care (ANC) utilization, remains underexplored. This study examines the association between internal migration and receiving antenatal care services among women aged 15–49 years using nationally representative cross-sectional data from the 2022 Bangladesh Demographic and Health Survey. This study analyzed 4,306 currently married women who had at least one live birth in the three years preceding the survey. Descriptive statistics, chi-square tests, and multivariable logistic regression models were employed to examine the association of migration status with receiving professional ANC and quality ANC visits. Our analysis shows that rural-to-urban migrants had the highest likelihood of receiving both professional ANC and quality ANC visits (68.5% and 38.2%, respectively). The adjusted odds ratios show that rural-to-urban migrants were significantly more likely to receive a professional ANC visit (AOR = 1.58, 95% CI: 1.11–2.24) than the urban residents, whereas urban-to-rural migrants were significantly less likely to receive that service (AOR = 0.74, 95% CI: 0.60–0.91) than their urban counterparts. A similar pattern was observed for the utilization of quality ANC (AOR = 0.73, 95% CI: 0.58–0.94) among urban-to-rural migrants. These findings indicate apparent inequalities among different groups of migrant women in their access to maternal healthcare services. Strengthening rural healthcare infrastructure and enhancing awareness about the importance of ANC could help ensure equitable access to maternal health care services in Bangladesh and other countries with similar contexts.

## Introduction

Migration has significant implications for the health and well-being of individuals, particularly for women and children. It plays an important role in shaping socioeconomic and healthcare dynamics experienced by individuals in their lifetimes, particularly women in their reproductive years (aged 15–49) [1]. Specifically, migrations between urban and rural areas in developing countries often lead to significant shifts in an individual’s socioeconomic status, access to essential services, and overall quality of life [2]. In many low- and middle-income countries, urban areas generally offer better healthcare infrastructure, skilled health professionals, and enhanced maternal health services. In contrast, rural areas frequently face challenges such as inadequate medical facilities, shortages of trained personnel, and social or cultural barriers that hinder healthcare utilization [3]. Consequently, women’s migration trajectories, whether moving from rural to urban settings, from urban to rural areas, or staying within the same settlement type, are closely linked to variations in access to and utilization of healthcare services [4]. The movement from one location to another not only changes the availability of services but also influences women’s health-seeking behavior, perceptions of care, and ability to access medical services [5,6].

According to the World Health Organization (WHO), as of 2022, approximately 86% of pregnant women globally receive at least one antenatal care (ANC) visit; however, only about 65% complete the recommended minimum of four ANC visits. The coverage varies significantly by region: in high-income countries, over 90% of women attend at least four ANC visits, whereas in low-income regions, including Sub-Saharan Africa and South Asia, coverage often falls below 50% [7,8]. For instance, in South Asia, the average rate of four or more ANC visits was approximately 49% in 2021 [7]. In Bangladesh, the 2022 Bangladesh Demographic and Health Survey (BDHS) reported that 41% of women had at least four ANC visits for their most recent live births in the two years preceding the survey, and only 21% received quality ANC [9]. Among reproductive-aged women (15–45 years old) in Bangladesh, 57% migrated from urban to rural areas, while 11% migrated from rural to urban areas in 2022 [9]. The effect of migration on healthcare utilization can vary based on both the direction of migration and the region’s level of development [10,11]. Again, the utilization of maternal healthcare services, particularly ANC, varies significantly across different migration patterns [12,13]. Studies indicate that women migrating to urban areas generally have better access to healthcare services, enabling them to receive the recommended four or more ANC visits [14–16]. In developed countries, internal migrants tend to experience smaller gaps in maternal health access due to relatively uniform healthcare systems [17–19].

Recent evidence worldwide underscores the critical role of migration in shaping health equity and healthcare disparities. Studies from South and Southeast Asia reveal that internal migration can either mitigate or exacerbate inequalities in maternal health, depending on the direction and destination of movement. For example, in India and Nepal, rural-to-urban migrants often get improved access to healthcare services; however, they still face administrative and affordability barriers [10,13,20,21]. Conversely, women who migrate to rural or peri-urban areas often experience a decline in healthcare access due to reduced service availability and infrastructure [22]. Similar disparities have been observed in Myanmar and Vietnam, where financial insecurity and limited transportation options limit rural women’s access to timely ANC [22–25]. These findings highlight that internal migration can act as either a protective or a risk factor for maternal healthcare service utilization, depending on the socio-economic and geographical context [11].

In Bangladesh, disparities in maternal healthcare utilization are even more pronounced [26]. Internal migration, especially among women, is common and driven by reasons such as seeking employment, gaining access to education, and marriage-related factors. However, this aspect of maternal healthcare utilization remains relatively understudied within the country. Comparative studies across neighboring South Asian nations offer compelling insights into how migration patterns shape maternal healthcare equity. For instance, research conducted in India, Nepal, and Pakistan demonstrated a clear association between internal migration and variations in healthcare accessibility and equity. In some instances, labour migration in India has affected utilization and equitable access to health care services [27]. In Nepal and Pakistan, migration of reproductive-aged women to urban areas has been associated with improved access to reproductive and maternal health care services [13,28]. Nevertheless, similar large-scale, nationally representative evidence is limited in the Bangladeshi context.

Bangladesh continues to face a high maternal mortality ratio of 123 deaths per 100,000 live births, alongside a relatively low ANC utilization rate, with fewer than half of women completing four visits [9]. Most existing research in Bangladesh has focused on economic inequality or rural–urban disparities in maternal healthcare service utilization rather than exploring the specific role of internal migration [16,29,30]. Nonetheless, internal migration has important implications for health equity. While it can help close the service gap for rural-to-urban migrants, it may worsen access issues for women moving from urban to rural areas, where healthcare facilities are often limited [5,12]. Understanding these migration-related dynamics is critical to designing equitable maternal healthcare policies.

Given the existing sharp differences in socioeconomic and health service provision between urban and rural areas, and the lack of relevant literature, the present study aims to examine the association between internal migration and ANC utilization among women of reproductive age in Bangladesh, using the most recent 2022 BDHS data. Because international migration is rare in this sample, it was excluded from the study. By analyzing differences in ANC utilization across migration categories, this research intends to contribute to the global discussion on maternal health equity and healthcare disparities, offering valuable evidence for policymakers and practitioners in other low- and middle-income countries with similar socioeconomic conditions.

## Materials and methods

### Data

This study employed a quantitative cross-sectional design, utilizing nationally representative data from the 2022 BDHS. The Demographic and Health Survey (DHS) Program has conducted similar nationally representative surveys in over 90 low- and middle-income countries, focusing on the demographic and health conditions of women and their families. The sampling framework for the 2022 BDHS is based on the Integrated Multi-Purpose Sampling Master Sample, which is derived from a complete list of enumeration areas (EAs) covering the whole country. This framework was prepared by the Bangladesh Bureau of Statistics for the 2011 population census of the People’s Republic of Bangladesh.

The 2022 BDHS survey used a two-stage stratified sampling method to select households. In the first stage, 675 EAs (237 in urban areas and 438 in rural areas) were selected with probability proportional to EA size. In the second stage of sampling, a systematic sample of 45 households per EA was selected to provide statistically reliable estimates of key demographic and health variables for urban and rural areas separately and for each of the eight divisions in Bangladesh. Ever-married women aged 15–49 (reproductive age) were considered eligible for individual interviews. The interviews were conducted between June 27 and December 12, 2022. Data were collected through structured, interviewer-administered questionnaires, developed and standardized by the DHS Program. The BDHS women’s questionnaire included modules on reproductive history, maternal and child healthcare, fertility preferences, and socioeconomic characteristics. The reliability and validity of the questionnaire have been established across multiple DHS rounds internationally, including in Bangladesh, through pre-testing, interviewer training, and strict data quality assurance protocols. More detailed information regarding the data collection process is available in the 2022 BDHS report [9].

**Fig 1:**
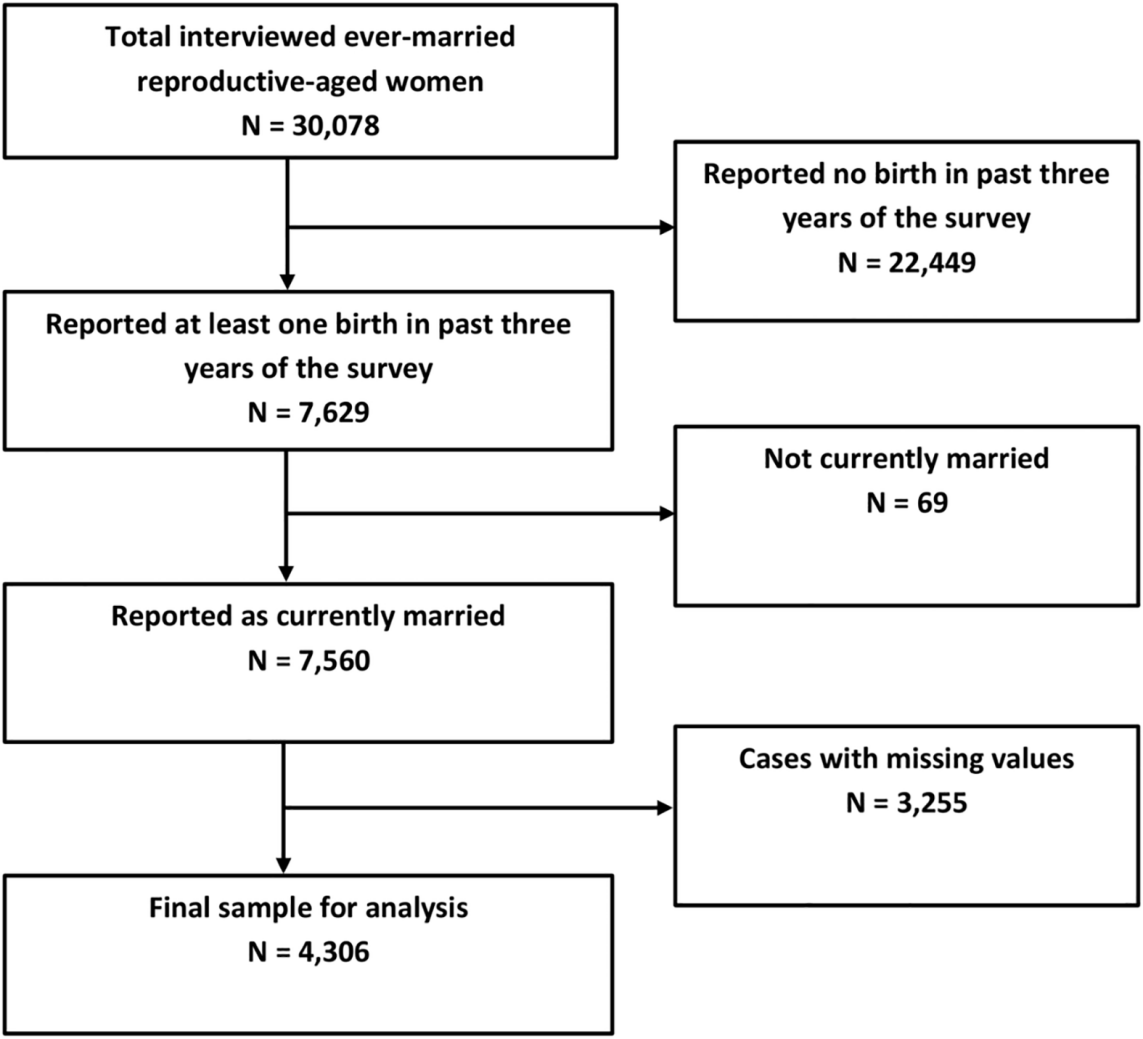
Flowchart of the sample selection process for the study.

The 2022 BDHS selected 30,330 households for interviewing, out of which 30,149 had occupants. Out of the occupied households, 30,018 were successfully interviewed, giving a response rate of almost 100%. From the interviewed households, 30,358 ever-married reproductive-aged women were found eligible for interviews, out of which 20,217 were eligible for the full questionnaire and 10,141 were eligible for the short questionnaire. Out of the eligible women, 30,078 were successfully interviewed using the full and short questionnaires with a response rate of 99%. The questionnaire is collated with the 2022 BDHS final report. We selected our analytical sample from those 30,078 women, and the sample selection process is illustrated in Fig 1. For our analysis, we used the “Individual Recode (IR) dataset”. Of the 30,078 ever-married reproductive-age women interviewed, 7,629 reported having at least one live birth in the three years preceding the survey. To meet the objectives of this study, the sample was further refined by excluding women who were not currently married (n = 69) and those with incomplete or missing information on key study variables (n = 3,255). After all the filtering, the analysis included 4,306 currently married women who had a live birth in the three years preceding the survey and had complete information on ANC utilization.

### Outcome variables

This study analyzed two key outcome variables: (i) the utilization of professional ANC visits and (ii) the utilization of ‘quality ANC’ services during the respondents’ most recent pregnancy. A woman was classified as having received professional ANC if she attended four or more ANC sessions, with at least one session conducted by a qualified healthcare provider. A qualified healthcare provider included a doctor, nurse, midwife, paramedic, family welfare visitor, community skilled birth attendant, medical assistant, or sub-assistant community medical officer [9]. Consistent with the definition provided in the 2022 BDHS, ‘quality ANC’ was defined as receiving at least four ANC visits–including at least one provided by a qualified healthcare provider–along with the five basic components of ANC at least once [9]. These basic components include measuring weight and blood pressure, blood and urine tests, and counseling on danger signs during pregnancy.

### Explanatory variables

Following existing literature, respondents’ migration status was categorized into five groups: urban residents (those who moved from urban-to-urban areas or were living in urban areas without migration), rural residents (those who moved from rural-to-rural areas or were living in rural areas without migration), urban-to-rural migrants, rural-to-urban migrants, and visitors [1,31]. The 2022 BDHS asked every respondent, “How long have you been living continuously in the current place of residence?” Respondents who answered “always” were classified as “non-migrant”, while those who provided a specific number of years as the period of living at their current location were identified as “migrants” if they had moved across district boundaries. Additionally, a follow-up question inquires about their previous place of residence prior to relocating. This information is utilized to create the aforementioned five categories. For example, a woman who indicated that her previous residence was in a rural area and her current residence was in an urban area was classified as a rural-to-urban migrant.

Based on the existing literature and availability of data in the 2022 BDHS, seven distinct background variables were selected for this study [13,31–33]. At the individual level, variables included the respondent’s current age (grouped as 15–19, 20–29, 30–39, and 40–49 years), age at first marriage, mass media exposure, birth order of index child, respondent’s education level, current working status, and religion. As a family-level variable, the respondent’s wealth index was included.

Age at first marriage was dichotomized as “<18 years” and “≥18 years,” distinguishing between child marriage and non-child marriage. Women were considered exposed to mass media if they had access to at least one of the following: newspapers, television, or radio. Birth order of index child refers to the birth order of the most recent child born within the three years prior to the survey, and this variable was categorized by first birth, second birth, third or more birth. Educational attainment was classified into five categories following the BDHS report: no education, incomplete primary education, complete primary education (completion of 5 years of schooling), incomplete secondary education, and complete secondary or higher education (completion of 10 years or more). Religion was categorized as Muslim and Non-Muslim. The non-Muslim group included those identifying themselves as Hindu, Buddhist, Christian, or followers of other minority religions recognized in Bangladesh. The respondent’s household wealth index was used as a proxy for socioeconomic status. It was constructed by the DHS Program using principal component analysis based on household ownership of consumer goods (e.g., televisions, bicycles, mobile phones), dwelling characteristics (e.g., flooring materials, water source, sanitation facilities), and other household assets. The variable wealth index was divided into five quintiles: poorest, poorer, middle, richer, and richest. The detailed methodology for constructing the wealth index is described elsewhere [34].

### Statistical analysis

A univariate analysis was performed to evaluate the background characteristics and the two outcome variables among the respondents. To assess the association of the two outcome variables with migration status and each of the other background characteristics, bivariate analysis using chi-squared (χ²) tests was performed. The chi-square test was suitable for examining associations between categorical variables and assessing the statistical significance of observed differences. Multivariable analyses involved multilevel binary logistic regression models to identify the association of migration streams with the two outcome variables while controlling for other relevant and available covariates. Because the outcome variables were binary, a binary logistic regression model was used. Findings of logistic regression are presented in terms of adjusted odds ratios accompanied by 95% confidence intervals (CIs). All analyses in this study were performed using a technique that accounts for the complex sampling design, which is crucial for accurately calculating significance levels and CIs. Data were analyzed using Stata version 17 (StataCorp LLC, College Station, TX, USA).

### Ethical considerations

This study is based on the data from the 2022 BDHS, conducted by the DHS Program. The DHS Program provides free, unrestricted access to survey data files for legitimate academic research. Permission was obtained from the DHS Program to access and analyze the 2022 BDHS dataset for this study. The data set was downloaded for research purposes from the DHS website on 20 December, 2023. The protocol for the 2022 BDHS was approved by both the ICF International Review Board ethics committee (project 2022–135-ICF) and the Bangladesh Medical Research Council (BMRC) [9]. Since this research employs de-identified secondary data, it does not require further ethical approval. The DHS Program adheres to stringent protocols to safeguard respondent privacy and confidentiality through de-identification of the respondents before publishing their data. The 2022 BDHS interviewed women aged 15–49 years. Prior to each interview of a respondent, the informed consent statement was loudly read before the respondent. A respondent was interviewed if she provided verbal consent for the interview in response to the consent statement reading. Comprehensive information regarding the DHS Program’s privacy protection measures for survey respondents can be found on their website.

## Results

### Background characteristics of the respondents

The background characteristics of the respondents are shown in Table 1. The migration status of respondents varied, with 21.1% urban residents, 12.8% rural residents, and the majority being urban-to-rural (U-R) migrants (53.5%). Among the respondents, 41.0% attended professional ANC visits, while 59.0% had fewer than four visits. Only 22.4% of the respondents received quality ANC services, while 77.6% did not.

**Table 1:** Background characteristics of the respondents (n = 4,306).

Background characteristics	Unweighted frequency	Weighted percentage (95% CI)
**Migrant status**		
Urban resident	1,126	21.1% (19.5%–22.8%)
Rural resident	500	12.8% (11.6%–14.1%)
Urban-to-rural migrant	2,095	53.5% (51.5%–55.5%)
Rural-to-urban migrant	212	4.4% (3.5%–5.6%)
Visitor	373	8.2% (7.2%–9.2%)
**Had professional ANC**		
No	2,488	59.0% (56.9%–61.2%)
Yes	1,818	41.0% (38.8%–43.1%)
**Quality ANC**		
Not taken	3,318	77.6% (75.9%–79.2%)
Taken	988	22.4% (20.8%–24.1%)
**Age group**		
15-19	603	14.9% (13.7%–16.2%)
20-29	2,591	59.8% (58.2%–61.3%)
30-39	1,053	24.1% (22.6%–25.6%)
40-49	59	1.2% (0.9%–1.6%)
**Age at first marriage**		
< 18	2,638	62.9% (61.1%–64.8%)
≥ 18	1,668	37.1% (35.2%–39.1%)
**Birth order**		
First	1,759	41.2% (39.5%–43.0%)
Second	1,489	33.7% (32.1%–35.3%)
Third or more	1,058	25.1% (23.6%–26.6%)
**Media exposure**		
Not exposed	1,749	40.4% (38.1%–42.7%)
Media exposed	2,557	59.6% (57.3%–61.9%)
**Education**		
No education	182	4.3% (3.6%–5.2%)
Incomplete primary	404	9.4% (8.3%–10.5%)
Complete primary	527	12.0% (10.9%–13.1%)
Incomplete secondary	1,704	41.5% (39.7%–43.4%)
Complete secondary or higher	1,489	32.8% (30.8%–34.9%)
**Work Status**		
Not working	3,395	77.9% (76.3%–79.4%)
Working	911	22.1% (20.6%–23.7%)
**Religion**		
Muslim	3,920	92.1% (90.2%–93.7%)
Non-Muslim	386	7.9% (6.3%–9.8%)
**Wealth quintile**		
Poorest	777	17.7% (16.2%–19.3%)
Poorer	852	20.3% (18.8%–22.0%)
Middle	870	21.1% (19.5%–22.8%)
Richer	891	20.9% (19.2%–22.7%)
Richest	916	20.0% (18.0%–22.3%)

The age distribution showed that most respondents (59.8%) were in the 20–29 age group, with a mere 1.2% aged 40–49. About 62.9% of women married before 18, while the rest married at 18 or later. In terms of birth order, 41.2% were first births, 33.7% were second births, and 25.1% were third or higher-order births. The majority of the respondents (59.6%) had mass media exposure, while 40.4% were not exposed. Regarding education, 4.3% had no formal education, 41.5% had incomplete secondary education, and 32.8% had completed secondary or higher education. Work status revealed that 77.9% of the women were not employed during the survey period, whereas 22.1% were working. The vast majority of respondents identified as Muslim (92.1%). Wealth was fairly evenly distributed, with around 20% in each wealth quintile, though the poorest group had a slightly lower percentage of 17.7.

### Association between ANC utilization, migration, and different background variables

**Fig 2:**
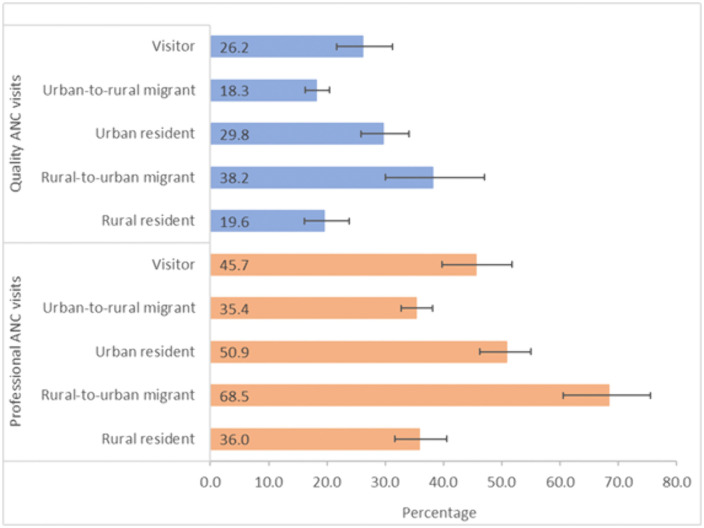
Percentage of women having professional ANC and quality ANC visits.

Fig 2 represents the changes in the ANC visit-seeking behaviors after migration. Only 36% and 19.6% of women in the rural areas were receiving, respectively, professional ANC and quality ANC visits. Among the women living in the urban areas, 50.9% and 29.8% were, respectively, receiving professional and quality ANC visits. Nevertheless, the percentages of receiving professional ANC and quality ANC visits almost doubled among the women who moved to urban areas from the rural areas. On the contrary, moving from urban to rural areas showed a remarkable negative influence on receiving both types of ANC visits.

The association between ANC utilization and various background variables is presented in Table 2. Women aged 40–49 had the highest percentage of professional ANC visits (46.8%), while the 15–19 age group had the lowest (35.4%). Compared to women who married before the age of 18, those who married at 18 or older had attended professional ANC visits (47.1%, p < 0.001) and received quality ANC (26.5%, p < 0.001) at higher rates. First and second-order births had similar ANC utilization rates, but third or higher-order births had the lowest professional ANC visits (35.5%) and the lowest prevalence of receiving quality ANC (19.7%). Women exposed to mass media had attended professional ANC visits (47.8% vs. 30.9%) and received quality ANC (27.1% vs. 15.5%) at higher rates than those who were not exposed. Women with completed secondary education or higher had the highest professional ANC visits (55.3%) and quality ANC (32.6%), while those with no education had the lowest utilization rates (27.0% and 13.9%, respectively). Women in the wealthiest quintile attended professional ANC visits at significantly higher rates (65.1%) compared to women in the poorest quintile (24.8%). Additionally, they received quality ANC (39.2%) nearly four times more frequently than the poorest group (10.3%).

**Table 2:** Utilization of professional ANC and quality ANC visits by migration and background characteristics of women in Bangladesh, 2022.

Background characteristics	Had professional ANC visits			Had quality ANC visits		
	Number	Percentage (95% CI)	p	Number	Percentage (95% CI)	p
**Migrant status**			p < 0.001			p < 0.001
Urban resident	465	50.6% (46.2%−55.0%)		274	29.8% (25.8%−34.1%)	
Rural resident	200	36.0% (31.6%−40.6%)		109	19.6% (16.1%−23.8%)	
Urban-to-rural migrant	824	35.4% (32.7%−38.1%)		425	18.3% (16.2%−20.4%)	
Rural-to-urban migrant	132	68.5% (60.6%−75.5%)		74	38.2% (30.0%−47.1%)	
Visitor	162	45.7% (39.7%−51.8%)		93	26.2% (21.7%−31.2%)	
**Age group**			p < 0.05			p < 0.001
15-19	229	35.4% (31.3%−39.7%)		100	15.5% (12.5%−18.9%)	
20-29	1066	41.0% (38.5%−43.5%)		598	23.0% (20.9%−25.1%)	
30-39	463	44.1% (40.4%−47.8%)		265	25.3% (22.3%−28.5%)	
40-49	25	46.8% (33.0%−61.2%)		12	22.5% (12.8%−36.2%)	
**Age at first marriage**			p < 0.001			p < 0.001
<18	1024	37.4% (35.1%−39.7%)		547	20.0% (18.1%−21.9%)	
>=18	759	47.1% (43.6%−50.5%)		428	26.5% (23.9%−29.3%)	
**Birth order**			p < 0.001			p = 0.084
First	776	43.3% (40.2%−46.3%)		419	23.3% (20.9%−25.9%)	
Second	619	42.2% (39.3%−45.2%)		341	23.3% (20.8%−25.8%)	
Third or more	388	35.5% (32.3%−39.0%)		215	19.7% (17.2%−22.6%)	
**Mass media exposure**			p < 0.001			p < 0.001
Not exposed	543	30.9% (28.3%−33.7%)		273	15.5% (13.5%−17.7%)	
Media exposed	1240	47.8% (45.1%−50.4%)		702	27.1% (24.9%−29.3%)	
**Education**			p < 0.001			p < 0.001
No education	50	27.0% (19.5%−36.1%)		26	13.9% (8.4%−22.2%)	
Incomplete primary	91	22.2% (17.9%−27.2%)		44	10.8% (7.8%−14.6%)	
Complete primary	169	32.5% (28.1%−37.3%)		82	15.8% (12.5%−19.8%)	
Incomplete secondary	683	37.8% (35.0%−40.6%)		357	19.8% (17.6%−22.1%)	
Complete secondary or higher	790	55.3% (51.9%−58.7%)		466	32.6% (29.6%−35.7%)	
**Work status**			p = 0.991			p = 0.503
Not working	1389	41.0% (38.7%−43.3%)		768	22.7% (20.9%−24.6%)	
Working	394	41.0% (37.1%−45.0%)		207	21.5% (18.5%−24.8%)	
**Religion**			p = 0.069			p = 0.904
Muslim	1625	40.5% (38.3%−42.8%)		897	22.4% (20.7%−24.2%)	
Non-Muslim	158	46.3% (40.5%−52.2%)		78	22.7% (18.5%−27.5%)	
**Wealth quintile**			p < 0.001			p < 0.001
Poorest	191	24.8% (21.4%−28.5%)		79	10.3% (8.2%−12.8%)	
Poorer	269	30.4% (27.0%−34.0%)		134	15.1% (12.5%−18.1%)	
Middle	332	36.2% (32.4%−40.3%)		180	19.7% (16.8%−22.8%)	
Richer	423	46.7% (43.0%−50.3%)		240	26.5% (23.2%−30.0%)	
Richest	568	65.1% (60.8%−69.1%)		342	39.2% (35.2%−43.4%)	

### Correlates of receiving professional ANC visits

**Fig 3:**
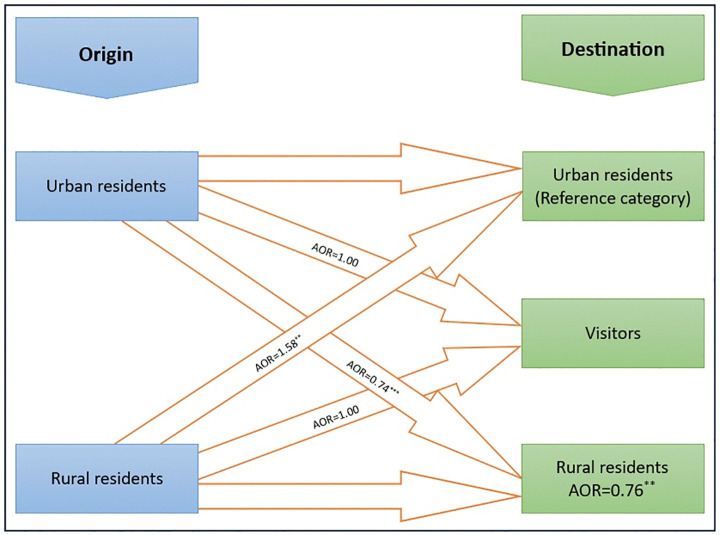
Adjusted odds ratio (AOR) of utilizing professional ANC by migration stream, Bangladesh, 2022. **Notes:** The AORs have been taken from Table 3. The urban residents in the destination include urban non-movers and those who moved to urban areas from other urban areas; the rural residents in the destination include rural non-movers and those who moved to rural areas from other rural areas. ***p < 0.01, **p < 0.05.

Adjusted odds of receiving professional ANC visits by migration stream have been presented in Fig 3. As it is seen from Fig 3, migration from rural to urban areas was associated with a 58% increase in the odds of receiving professional ANC visits (AOR: 1.58, 95% CI: 1.11–2.24, p < 0.05). Compared to urban residents, both rural residents (AOR: 0.76, 95% CI: 0.58–0.99, p < 0.05) and urban-to-rural migrants (AOR: 0.74, 95% CI: 0.60–0.91, p < 0.01) had significantly lower odds of receiving professional ANC visits and, while rural-to-urban migrants had significantly higher odds (AOR: 1.58, 95% CI: 1.11–2.24, p < 0.05).

**Table 3:** Estimates of the adjusted odds ratio of utilizing professional ANC and quality ANC in Bangladesh, 2022.

Variables	Adjusted Odds Ratio of professional ANC (95% CI)	Adjusted Odds Ratio of Quality ANC (95% CI)
**Migrant status (Ref: Urban resident)**		
Rural resident	0.76 (0.58–0.99)**	0.80 (0.58–1.10)
Urban-to-rural migrant	0.74 (0.60–0.91)***	0.73 (0.58–0.94)**
Rural-to-urban migrant	1.58 (1.11–2.24)**	1.10 (0.69–1.74)
Visitor	1.00 (0.74–1.35)	1.04 (0.76–1.42)
**Age group (Ref: 15–19)**		
20-29	1.23 (0.95–1.59)	1.65 (1.20–2.28)***
30-39	1.56 (1.11–2.18)**	2.05 (1.36–3.09)***
40-49	1.77 (0.81–3.84)	1.74 (0.74–4.10)
**Age at first marriage (Ref: < 18)**		
>=18	0.98 (0.81–1.18)	0.92 (0.75–1.13)
**Birth order (Ref: First)**		
Second	0.88 (0.73–1.07)	0.85 (0.69–1.05)
Third or more	0.77 (0.61–0.98)**	0.82 (0.61–1.09)
**Media exposure (Ref: Not exposed)**		
Exposed	1.40 (1.20–1.64)***	1.39 (1.14–1.70)***
**Education (Ref: No education)**		
Incomplete primary	0.77 (0.46–1.28)	0.73 (0.38–1.40)
Complete primary	1.26 (0.78–2.04)	1.09 (0.58–2.07)
Incomplete secondary	1.41 (0.91–2.19)	1.29 (0.72–2.30)
Complete secondary or higher	2.05 (1.30–3.23)***	1.81 (1.00–3.29)
**Work Status (Ref: Not working)**		
Working	1.15 (0.96–1.38)	1.04 (0.84–1.28)
**Religion (Ref: Muslim)**		
Non-Muslim	1.24 (0.92–1.67)	0.95 (0.72–1.26)
**Wealth quintile (Ref: Poorest)**		
Poorer	1.10 (0.87–1.39)	1.31 (0.95–1.80)
Middle	1.27 (0.97–1.66)	1.61 (1.18–2.21)***
Richer	1.76 (1.36–2.29)***	2.16 (1.56–3.00)***
Richest	2.90 (2.16–3.90)***	3.02 (2.13–4.29)***

Notes: Reference category is denoted by (Ref). Significance: ***p < 0.01, **p < 0.05.

Table 3 shows the correlates of receiving professional ANC visits, as identified through binary logistic regression analysis. The results are presented in terms of adjusted odds ratio along with a 95% CI. Age played a significant role, with women aged 30–39 having 1.56 times higher odds (p < 0.05) of receiving professional ANC visits compared to those in the 15–19 age group. Birth order negatively impacted ANC visits, with women having three or more children showing significantly lower odds (AOR: 0.77, p < 0.05). Media exposure was associated with a 40% increase in the odds of receiving professional ANC visits (AOR: 1.40, p < 0.001). Education had a powerful influence, with women who completed secondary education having more than twice the odds of attending professional ANC visits (AOR: 2.05, p < 0.01) compared to those with no education. Wealth emerged as a critical determinant, as the richest women were nearly three times more likely (AOR: 2.90, p < 0.001) to attend professional ANC visits compared to the poorest group.

### Correlates of receiving quality ANC visits

**Fig 4:**
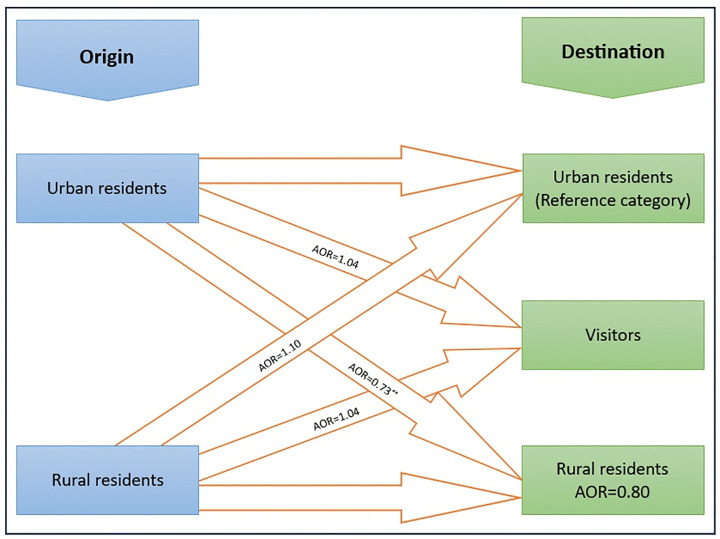
Adjusted odds ratio (AOR) of utilizing quality ANC by migration stream, Bangladesh, 2022. **Notes:** The AORs have been taken from Table 3. The urban residents in the destination include urban non-movers and those who moved to urban areas from other urban areas; the rural residents in the destination include rural non-movers and those who moved to rural areas from other rural areas. **p < 0.05.

Fig 4 presents the adjusted odds of receiving quality ANC visits by internal migration streams. In using quality ANC, only the migration from urban to rural areas showed a significant influence on receiving quality ANC visits. The odds of receiving quality ANC for the urban-to-rural migrants was 27% lower than that for the urban residents (AOR = 0.73, 95% CI: 0.58–0.94, p < 0.05).

Table 3 also shows the correlates of quality ANC utilization, as identified through binary logistic regression analysis. The results are also presented in terms of adjusted odds ratios along with 95% CIs. Older women were more likely to receive quality ANC, with ages 20–29 (AOR: 1.65, p < 0.001) and 30–39 (AOR: 2.05, p < 0.001) showing significantly higher odds than the 15–19 age group. Media exposure played a significant role, with exposed women 1.39 times more likely (AOR: 1.39, p < 0.001) to receive quality ANC. Wealth remained a crucial factor, as the richest women had three times higher odds of receiving quality ANC (AOR: 3.02, p < 0.001) than the poorest group.

## Discussion

This study primarily examined the association between internal migration and receiving ANC visits among currently married women aged 15–49 years in Bangladesh. The findings showed significant differences in ANC visits among women with different migration statuses. Rural-to-urban migrants had the highest rates of receiving professional ANC visits and quality ANC visits, while urban-to-rural migrants had the lowest ANC visit rate. Specifically, 68.5% and 38.2% of women who migrated from rural to urban areas, respectively, had professional and quality ANC visits, while the percentages of having professional and quality ANC visits among those who migrated from urban to rural areas were, respectively, 35.4% and 18.3%. The probabilities of receiving professional ANC and quality ANC visits for a woman on average almost doubled when she moved to an urban area from a rural area. Besides migration status, other significant factors associated with ANC visits included respondents’ age, media exposure, education, and wealth quantile.

The increased ANC utilization among rural-to-urban migrants aligns with findings from other developing countries such as India, Nepal, and Vietnam, where migrants relocating to urban areas benefit from better access to healthcare infrastructure, skilled healthcare service providers, and improved transportation systems [35–37]. In contrast, migrants relocating to rural areas often have reduced service availability and lower-quality facilities, as observed in Myanmar and Pakistan [23,28]. These patterns indicate that the direction of migration plays a crucial role in shaping maternal healthcare utilization in the settings with limited resources. A study comparing health service needs found that rural-to-urban migrants had higher healthcare needs (11.99%) than urban residents (10.47%), potentially leading to increased utilization of available services [38]. Extensive healthcare service availability and improved transportation and communication systems in urban areas contributed to the higher uptake of ANC services by rural-to-urban migrants [12].

Differences in ANC service uptake among migrants, however, cannot be solely attributed to disparities in service infrastructure between rural and urban areas. Migration itself introduces unique challenges that can significantly affect healthcare-seeking behavior. For instance, newly arrived migrants may lack information about available health facilities, face language or cultural barriers, or experience social isolation that may limit access to services. Migrant women, especially those moving to rural areas, may have less autonomy in healthcare decision-making and restricted mobility due to conservative social norms in some societies [39]. On the other hand, rural-to-urban migrants may initially show higher healthcare utilization, not only because of greater availability but also because of increased health awareness and motivation to access urban services after experiencing improved healthcare environments. These psychosocial and cultural dimensions reveal the complexity of the relationship between migration and ANC utilization, which is shaped by both contextual and individual influences. Prior studies concluded that rural areas in Bangladesh lack adequate health care service delivery, which in turn leads rural women to use healthcare services less frequently [3,16]. The poor utilization of ANC services by the urban-to-rural migrants mirrors conditions observed in other low-income countries, such as Nepal and Cambodia, where rural health systems remain under-resourced and overburdened [40,41]. Rural areas in Bangladesh face similar challenges, including shortages of qualified healthcare providers, inadequate transportation networks, and limited community-level health education [3,16]. Addressing these systemic weaknesses is essential for reducing healthcare inequities and achieving universal access to quality maternal care.

In addition to women’s geographic movements, other factors showed statistically significant association with receiving ANC visits, including age, exposure to media, education, and household wealth status. Women aged 20–29 years were more likely to attend ANC sessions than the teens. Women exposed to media had higher odds of receiving ANC services than those not exposed to media. Women with complete secondary or higher education received higher odds of attending professional ANC sessions than those who had no education. The household wealth status showed a positive relationship with the odds of receiving ANC visits. All these results are analogous to the findings of past studies [6,13,15,28,42,43].

### Program and policy implications

Findings of this study could be productively used in formulating maternal health policy. Strengthening rural healthcare infrastructure and ensuring an equitable distribution of skilled healthcare providers could narrow the service gap between rural and urban areas. Establishing community-based awareness programs targeting migrant and rural women could increase understanding of the importance of ANC utilization and other available healthcare services. Expanding mobile health (mHealth) initiatives and deploying trained community health workers may also help overcome geographic and social barriers to accessing ANC services [44–46]. In addition, incorporating migration-sensitive strategies such as health registration systems that allow women to access services regardless of residence could better integrate migrants into existing healthcare frameworks [47]. These measures would improve maternal health outcomes and contribute to broader goals of health equity and social inclusion in Bangladesh and other countries with similar socioeconomic settings.

### Strengths

This study has several significant strengths. Unlike some earlier studies, we employed the complex survey design that incorporates sample weights, clustering, and stratification, thereby making our results more accurate and nationally representative [38]. As the BDHS data are hierarchical in nature, DSH strongly recommends using an analysis technique that accounts for the complex survey design [48]. We utilized the most recent 2022 BDHS data, which marks the first survey conducted post-COVID-19 pandemic. Since the pandemic changed healthcare utilization patterns, particularly regarding maternal care, our findings provide a more current reflection of the situation compared to previous studies [49,50]. Also, very few studies in Bangladesh have focused on the link between internal migration and antenatal care. While many other studies have addressed various types of maternal services, our focus narrows to antenatal care visits, enabling a deeper exploration of this area. Hence, the findings of this study are likely to contribute to the growing body of evidence on the influence of internal migration on maternal healthcare utilization in Bangladesh.

### Limitations

Reporting the limitations of a study is also imperative. First, the cross-sectional nature of the BDHS data limits causal interpretation; the observed associations cannot confirm temporal relationships between migration and ANC utilization. Second, information on ANC visits was self-reported and may be subject to recall bias, particularly among women reporting births up to three years before the survey. Some factors such as the characteristics of migration duration and reasons of migration were similar to that of migration status. These factors also had one subcategory in common, which is visitors. Therefore, migration duration and reasons of migration were not included in the model with the migration status that was our primary exposure variable. Besides receiving ANC visits, some other services are also essential for maintaining the good health of the mother and child. Independent studies investigating the influences of urban-rural migration on the uptake of other crucial maternal healthcare services (such as receiving assistance from a trained birth attendant at delivery) could be productive.

## Conclusions

The current study provides critical insights into women’s ANC visit-seeking behavior and urban-rural differences in healthcare service provisions. Our findings show that women who migrated from rural to urban areas had significantly higher odds of attending ANC sessions, reflecting better maternal health care provision in the urban areas. Therefore, improvement in maternal healthcare service delivery in rural areas may lead to a significant increase in the utilization of ANC visits in rural areas. Future research should explore the social and psychological dimensions of migration that influence maternal health, such as women’s autonomy, community support, and the role of informal social networks in health-seeking behavior, and also show the interaction effects, particularly between migration status and socioeconomic variables such as education and wealth, to better understand intersectional inequalities in ANC access. Expanding this evidence base will be essential for designing migration-sensitive health policies and achieving equitable maternal healthcare in Bangladesh and other low- and middle-income countries facing similar challenges.
