# Supplementary material for: Influence of internal migration on antenatal care utilization in Bangladesh: Findings from a nationally representative cross-sectional survey
Source: PLoS One. 2026 Jun 26;21(6):e0345817. doi: 10.1371/journal.pone.0345817 (PMC13308873; doi:10.1371/journal.pone.0345817)
Supplement: Attachment — Submitted filename: Response to Reviewers.docx [file pone.0345817.s002.docx]

**Journal Requirements**

When submitting your revision, we need you to address these additional requirements.

1. Please ensure that your manuscript meets PLOS ONE's style requirements, including those for file naming. The PLOS ONE style templates can be found at <https://journals.plos.org/plosone/s/file?id=wjVg/PLOSOne_formatting_sample_main_body.pdf> and <https://journals.plos.org/plosone/s/file?id=ba62/PLOSOne_formatting_sample_title_authors_affiliations.pdf>

**Authors’ response**

Manuscript have been formatted as per PLOS ONE’s guidelines.

2. Your ethics statement should only appear in the Methods section of your manuscript. If your ethics statement is written in any section besides the Methods, please delete it from any other section.

**Authors’ response**

This guideline has been followed.

 3. If the reviewer comments include a recommendation to cite specific previously published works, please review and evaluate these publications to determine whether they are relevant and should be cited. There is no requirement to cite these works unless the editor has indicated otherwise.

**Authors’ response**

We have followed this guideline.

**Additional Editor Comments**

**Authors’ overall response**

The authors would like to thank the editor and reviewers for providing insightful suggestions. The authors consider all the suggestions are very helpful in improving the explanation and readability of the manuscript. Therefore, the authors have addressed all the reasonable suggestions. Besides these, the authors have revised whole manuscript again to improve the sentences and explanations in the manuscript. We appreciate the time and considerations of the editor and reviewers.

**Suggestions for improvement:**

1. The introduction could be bolstered by incorporating more recent global literature on migration and maternal healthcare, including studies from other South Asian countries.

**Authors’ response**

Thank you for this insightful comment. We have improved our introduction section as per your suggestion. Please see the lines in the file “Revised Manuscript with Track Changes”: 110-122.

2. The authors should discuss potential confounders that may not have been fully controlled, including migration duration and reasons for migration.

**Authors’ response**

Thank you for raising this important point. Our main focus was to examine the influence of migration status on ANC utilization. Migration status is the counterpart of migration duration and reasons of migration. Migration status, migration duration, and migration reasons have one category (visitors) in common. Therefore, these variables are very similar in terms of their characteristics. In case of reasons of migration, about 63% of the total selected cases (4306) migrated for marrying a man living in another area, 22% of the total selected cases were non-migrant, 3.4% (or 119 cases) of the total cases migrated for employment, 0.4% (or 17 cases) of the total cases migrated for education, etc. These statistics clearly show that reasons of migration will create a problem of insufficient cell frequency that shall make the drawing of statistical conclusion very difficult. These were some reasons of excluding these variables from the analysis. Nevertheless, based on your suggestion, we added explanatory text in the limitations section noting that these variables may act as unmeasured confounders.

Please see the lines in the file “Revised Manuscript with Track Changes”: 577-580.

3. The study could be strengthened by exploring interaction effects, for example, between migration status and wealth or education level.

**Authors’ response**

Thank you for this useful suggestion. We explored interaction effects between migration status and both wealth and education. However, the results of the analyses were not meaningful or interpretable. We did not include the interaction effects for the following reasons.

The addition of the interaction effects between migration and education spoilt the model, and all of the interaction effects and migration stream-categories became statistically non-significant in the model. See the Table 1 presenting only the adjusted odds ratios (AOR) of migration and interactions (AORs of other variables have not been presented to make the table concise)

**Table 1. Interaction Effects of Migration Stream and Educational Attainment on Utilization of 4^+^ ANC Visits and Quality ANC Services**

| **Variables** | **Adjusted Odds Ratio of 4^*^ professional ANC (95% CI)** | **Adjusted Odds Ratio of Quality ANC (95% CI)** |
| --- | --- | --- |
| **Main effects** |  |  |
| **Migrant status (Ref: Urban resident)** |  |  |
| Rural resident | 0.43 (0.11–1.61) | 0.30 (0.04–2.01) |
| Urban–rural migrant | 0.88 (0.33–2.35) | 0.70 (0.19–2.55) |
| Rural–urban migrant | 0.65 (0.14–2.97) | 0.67 (0.09–4.75) |
| Visitor | 0.71 (0.08–5.95) | 1.64 (0.16–17.28) |
| **Education (Ref: No education)** |  |  |
| Incomplete primary | 0.60 (0.23–1.55) | 0.69 (0.20–2.34) |
| Complete primary | 0.87 (0.31–2.42) | 0.92 (0.24–3.44) |
| Incomplete secondary | 1.37 (0.61–3.07) | 0.95 (0.30–2.97) |
| Complete secondary/higher | 2.19 (0.94–5.09) | 1.78 (0.56–5.65) |
| **Interaction effects** |  |  |
| Rural × incomplete primary | 1.94 (0.40–9.47) | 1.73 (0.18–16.36) |
| Rural × complete primary | 2.40 (0.49–11.74) | 2.74 (0.32–23.80) |
| Rural × incomplete secondary | 1.87 (0.49–7.20) | 3.64 (0.54–24.80) |
| Rural × complete secondary/higher | 1.56 (0.39–6.25) | 1.60 (0.23–11.15) |
| U–R × incomplete primary | 1.23 (0.37–4.10) | 1.18 (0.27–5.20) |
| U–R × complete primary | 1.32 (0.39–4.45) | 1.15 (0.25–5.21) |
| U–R × incomplete secondary | 0.83 (0.30–2.25) | 1.31 (0.35–4.90) |
| U–R × complete secondary/higher | 0.68 (0.24–1.92) | 0.91 (0.24–3.42) |
| R–U × incomplete primary | 1.74 (0.19–15.85) | 0.56 (0.04–8.69) |
| R–U × complete primary | 3.38 (0.46–25.07) | 0.93 (0.07–12.57) |
| R–U × incomplete secondary | 2.19 (0.48–10.09) | 2.46 (0.30–20.15) |
| R–U × complete secondary/higher | 3.02 (0.56–16.40) | 1.69 (0.23–12.72) |
| Visitor × incomplete primary | 2.22 (0.19–25.74) | 0.56 (0.03–10.28) |
| Visitor × complete primary | 1.56 (0.14–17.91) | 1.10 (0.08–15.89) |
| Visitor × incomplete secondary | 1.26 (0.14–11.29) | 0.66 (0.06–7.65) |
| Visitor × complete secondary/higher | 1.51 (0.17–13.13) | 0.60 (0.06–6.47) |

When the interaction effects of migration and wealth index were introduced in the model that also produced the inconsistent and meaningless results. Relevant results of this model have been presented in Table 2 (AORs of other variables have not been presented to make the table concise). For example, introduction of the interaction effects to the model unusually increased the odds ratio of receiving 4^+^ professional ANC for urban-rural migrants to 24.12. Besides these, the AORs of all the interaction terms became less than one (1), which is logically inconsistent. For example, when rural people moved to urban areas, they were more likely to have 4^+^ professional ANC than the urban residents, that is their AOR was >1. In the same way, if a richest rural person moved to urban area, his odds of receiving 4^+^ professional ANC should have been more than one or more than those who were poor urban residents. But Table 2 shows that the odds of receiving 4^+^ professional ANC by the richest rural persons who moved to urban areas (AOR: 0.07, 95% CI: 0.01–0.61, p<0.05) was less than those who were poor urban residents. Most of the AORs presented in the Table 2 could be logically inconsistent in the same way.

**Table 2: Interaction Effects of Migration Stream and wealth index on Utilization of 4^+^ ANC Visits and Quality ANC Services**

| **Variable** | **Adjusted Odds Ratio of ≥4 professional ANC (95% CI)** | **Adjusted Odds Ratio of Quality ANC (95% CI)** |
| --- | --- | --- |
| **Main effects** |  |  |
| **Migrant status (Ref: Urban resident)** | 1.30 (0.60–2.82) | 3.08 (0.91–10.38) |
| Rural resident | 1.38 (0.72–2.64) | 2.99 (1.05–8.51)** |
| Urban–rural migrant | 24.12 (3.05–191.01)*** | 4.16 (0.41–42.64) |
| Rural–urban migrant | 2.65 (1.01–6.97)** | 4.26 (1.05–17.38)** |
| **Wealth Index (ref: poorest)** |  |  |
| Poorer | 2.43 (1.27–4.64)*** | 6.44 (1.85–22.50)*** |
| Middle | 1.89 (0.90–3.97) | 5.23 (1.78–15.39)*** |
| Richer | 2.90 (1.54–5.46)*** | 7.63 (2.65–21.95)*** |
| Richest | 6.06 (3.05–12.05)*** | 11.31 (3.89–32.89)*** |
| **Interaction: Migration Stream × Wealth Status (ref: Non-migrant × Poor)** |  |  |
| Rural Resident × Poor | 0.51 (0.21–1.23) | 0.20 (0.05–0.88)** |
| Rural Resident × Middle | 0.80 (0.31–2.08) | 0.27 (0.07–0.98)** |
| Rural Resident × Richer | 0.64 (0.26–1.57) | 0.22 (0.06–0.82)** |
| Rural Resident × Richest | 0.32 (0.13–0.83)** | 0.16 (0.04–0.59)*** |
| U-R × Poor | 0.43 (0.21–0.87)** | 0.17 (0.05–0.63)*** |
| U-R × Middle | 0.65 (0.29–1.46) | 0.29 (0.09–0.91)** |
| U-R × Richer | 0.57 (0.28–1.16) | 0.26 (0.09–0.80)** |
| U-R × Richest | 0.37 (0.17–0.80)** | 0.24 (0.08–0.75)** |
| R-U × Poor | 0.20 (0.01–2.80) | 0.39 (0.02–6.21) |
| R-U × Middle | 0.04 (0.00–0.47)*** | 0.38 (0.03–5.71) |
| R-U × Richer | 0.05 (0.01–0.48)*** | 0.22 (0.02–2.64) |
| R-U × Richest | 0.07 (0.01–0.61)** | 0.28 (0.03–3.08) |
| Visitors × Poor | 0.29 (0.10–0.84)** | 0.19 (0.04–1.03) |
| Visitors × Middle | 0.90 (0.30–2.73) | 0.38 (0.09–1.63) |
| Visitors × Richer | 1.34 (0.48–3.77) | 0.50 (0.12–2.04) |
| Visitors × Richest | 0.85 (0.30–2.45) | 0.40 (0.09–1.72) |

Note: Both models are adjusted for migration duration, migration cause age group, age at first marriage, birth order, media exposure, education, work status, religion, and wealth quintile. Reference category is denoted by (ref). Significance: ***p < 0.01, **p < 0.05.

4. More visual representations of data, such as maps indicating migration flows and their relation to ANC coverage, may enhance reader understanding.

**Authors’ response**

Thank you for the suggestion. The BDHS does not provide necessary geographic location indicating individual’s migration from one type of area to other types; therefore, we could not make map to show the migration flows and the changes in the ANC use rates. Nevertheless, we have made flow charts to make it is easier to understand the migration stream and the differences in the odds of receiving ANC services. Please see the figures 3 and 4 in the file “Revised Manuscript with Track Changes”.

5. Provide more detailed discussion on potential mechanisms behind the observed differences in ANC utilization beyond infrastructure availability, particularly focusing on social and cultural barriers that migrant women might face.

**Authors’ response**

Thank you for this important suggestion. We expanded the Discussion to include sociocultural and psychosocial mechanisms that affect migrant women’s healthcare-seeking behaviour. Please see the lines in “Revised Manuscript with Track Changes”: 507-514.

6. Consider expanding the limitations section to address potential recall bias in survey responses and the cross-sectional design's restricted ability to infer causality.

**Authors’ response**

We added a detailed paragraph addressing these concerns. Please see the lines in “Revised Manuscript with Track Changes”: 573-577.

7. The conclusion could be more forward-looking, suggesting areas for future research on maternal health and migration.

**Authors’ response**

We revised the Conclusion to include directions for future research. Please see following lines in “Revised Manuscript with Track Changes”: 599-606.

--------------------------------------------------------------------------------------------------------------------------

[Note: HTML markup is below. Please do not edit.]

**Reviewers' comments:**

Reviewer's Responses to Questions

**Comments to the Author**

1. Is the manuscript technically sound, and do the data support the conclusions?

The manuscript must describe a technically sound piece of scientific research with data that supports the conclusions. Experiments must have been conducted rigorously, with appropriate controls, replication, and sample sizes. The conclusions must be drawn appropriately based on the data presented.

Reviewer #1: Yes

Reviewer #2: Yes

2. Has the statistical analysis been performed appropriately and rigorously?

Reviewer #1: Yes

Reviewer #2: Yes

3. Have the authors made all data underlying the findings in their manuscript fully available?

The [PLOS Data policy](http://track.editorialmanager.com/CL0/http:%2F%2Fwww.plosone.org%2Fstatic%2Fpolicies.action%23sharing/1/010f0199b7a813b1-6efed900-e553-4ea9-a583-4d55ad0e899e-000000/-Sty_DFBfFCyBlB_LMi24SrPuzMfTNXuTcL__YBa3z8=231) requires authors to make all data underlying the findings described in their manuscript fully available without restriction, with rare exception (please refer to the Data Availability Statement in the manuscript PDF file). The data should be provided as part of the manuscript or its supporting information, or deposited to a public repository. For example, in addition to summary statistics, the data points behind means, medians and variance measures should be available. If there are restrictions on publicly sharing data—e.g. participant privacy or use of data from a third party—those must be specified.

Reviewer #1: Yes

Reviewer #2: Yes

4. Is the manuscript presented in an intelligible fashion and written in standard English?

PLOS ONE does not copyedit accepted manuscripts, so the language in submitted articles must be clear, correct, and unambiguous. Any typographical or grammatical errors should be corrected at revision, so please note any specific errors here.

Reviewer #1: No

Reviewer #2: Yes

5. Review Comments to the Author

Please use the space provided to explain your answers to the questions above. You may also include additional comments for the author, including concerns about dual publication, research ethics, or publication ethics. (Please upload your review as an attachment if it exceeds 20,000 characters)

**Reviewer #1**

Thank you for the opportunity to review this paper. The idea of the paper is good but more work needs to go into this paper. My suggestions are as follows:

1. The authors should revise the abstract and title for clarity, structure, and accuracy (focusing better on the methodology they used to develop findings in the research work)

**Authors’ response**

The abstract and title were revised to improve clarity, structure, and methodological transparency. Please see the title and abstract in “Revised Manuscript with Track Changes.”

2. The "Introduction" should be developed, modified, rearranged and divided into some paragraphs based on scientific writing. To strengthen the paper I would suggest consideration to expand the introduction and bring more from literature about the idea of this paper (health equity & healthcare disparities) and shortening the paragraphs; that would make a stronger contribution to the international audiences.

**Authors’ response**

Thank you for this suggestion. The Introduction was reorganized into multiple focused paragraphs, shortened for clarity, and strengthened with recent global and South Asian evidence on health equity, healthcare disparities, and migration. Please see the introduction’s track changed portions in “Revised Manuscript with Track Changes.”

3- The authors should explain more about the type of research (quantitative or Qualitative or Mix Method), in the beginning of "material and method"

**Authors’ response**

To address this suggestion, we have added following lines in “Revised Manuscript with Track Changes”: 189.

4- the authors should mention and justify why they have used the type of statistic test, and mention the statistical software they have employed in this study.

**Authors’ response**

Thank you. We clarified the analytical justification and software: Please see following lines in “Revised Manuscript with Track Changes”: 285-305.

5. The method of extracting and analyzing data from interviews and also validity and reliability of tools is not mentioned.

**Authors’ response**

We have addressed these suggestions in following lines in “Revised Manuscript with Track Changes.”: 206-211.

6. The authors should mention the questionnaire they used for interview.

**Authors’ response**

We have provided information about the questionnaire in the lines in “Revised Manuscript with Track Changes.”: 222

7. The "result" should be structured based on the main goal (question) & specific goals of the study.

**Authors’ response**

We have tried to address this suggestion by breaking the result section into two parts namely **Correlates of receiving four or more professional ANC** and **Correlates of receiving Quality ANC visits.** In these sections, we further subdivided the results. For example, as our main focus was on the association between internal migration and ANC utilization, we have presented these results with flowcharts at the beginning of each section. Please see the **Correlates of receiving four or more professional ANC** and **Correlates of receiving Quality ANC visits** sections in “Revised Manuscript with Track Changes.”.

8. Tables are too long and can be divided into 2 or 3 tables to be better for conveying the results of the research.

**Authors’ response**

We appreciate the suggestion. Our main focus was on the association between internal migration and ANC use. To make our main results easier to understand, we have made to figures (fig 3 and fig 4). These figures are expected to help readers easily understand the association between migration and ANC utilization. Besides these, we decided to retain the structure of our original table because we think that splitting them would reduce comparability among the results for the two outcome variables.

9. In 'Discussion" the authors should mention the situation of rural healthcare for women in countries with similar context to Bangladesh.

**Authors’ response**

Thank you for this valuable suggestion. In response to this suggestion, we expanded the Discussion section by adding comparative evidence from countries with similar healthcare challenges such as India, Nepal, Myanmar, Vietnam, Cambodia, and Pakistan. Please see following lines in “Revised Manuscript with Track Changes.”: 493-497 & 526-532.

**Reviewer #2**

The paper addresses an important topic of ANC care and Quality ANC care and the service delivery options in rural and urban settings. I hope that authors can comment on whether migrants more urban to rural settings are worse off than a rural resident. There could be a little more discussion on whether the differences we observe are just because of the service quality and access in rural vs urban settings or do the migrants have a specific experience. The program implications could be strengthened

**Authors’ response**

Thank you for raising this. We clarified in the Discussion that urban-to-rural migrants show significantly lower ANC utilization than both urban residents and rural non-migrants. We made the explanation of this difference clearer. We strengthened the Discussion by explaining that both structural differences and migrant-specific factors contribute to those disparities. We developed a more robust set of programme recommendations as well. Please see following lines in “Revised Manuscript with Track Changes.”: 507-519 & 542-553.

6. PLOS authors have the option to publish the peer review history of their article ([what does this mean?](https://track.editorialmanager.com/CL0/https:%2F%2Fjournals.plos.org%2Fplosone%2Fs%2Feditorial-and-peer-review-process%23loc-peer-review-history/1/010f0199b7a813b1-6efed900-e553-4ea9-a583-4d55ad0e899e-000000/lj6_oxooCpaDyVqTHI23VdBN6pD65OD4PkxSvHnx-WE=231)). If published, this will include your full peer review and any attached files.


If you choose “no”, your identity will remain anonymous but your review may still be made public.


**Do you want your identity to be public for this peer review?** For information about this choice, including consent withdrawal, please see our [Privacy Policy](https://track.editorialmanager.com/CL0/https:%2F%2Fwww.plos.org%2Fprivacy-policy/1/010f0199b7a813b1-6efed900-e553-4ea9-a583-4d55ad0e899e-000000/H8G8zSGhYRDa-LqwAy61q5OyaiRymhXaseYMh_Sk3H0=231).

Reviewer #1: No

Reviewer #2: No

[NOTE: If reviewer comments were submitted as an attachment file, they will be attached to this email and accessible via the submission site. Please log into your account, locate the manuscript record, and check for the action link "View Attachments". If this link does not appear, there are no attachment files.]

While revising your submission, please upload your figure files to the Preflight Analysis and Conversion Engine (PACE) digital diagnostic tool, [https://pacev2.apexcovantage.com/](https://track.editorialmanager.com/CL0/https:%2F%2Fpacev2.apexcovantage.com%2F/1/010f0199b7a813b1-6efed900-e553-4ea9-a583-4d55ad0e899e-000000/2ckIjAWnjjtsnPiC4L8zB2BF9Wuydws0pxXaGjTlObE=231). PACE helps ensure that figures meet PLOS requirements. To use PACE, you must first register as a user. Registration is free. Then, login and navigate to the UPLOAD tab, where you will find detailed instructions on how to use the tool. If you encounter any issues or have any questions when using PACE, please email PLOS at [figures@plos.org](mailto:figures@plos.org). Please note that Supporting Information files do not need this step.
