# Supplementary material for: Influence of internal migration on antenatal care utilization in Bangladesh: Findings from a nationally representative cross-sectional survey
Source: PLoS One. 2026 Jun 26;21(6):e0345817. doi: 10.1371/journal.pone.0345817 (PMC13308873; doi:10.1371/journal.pone.0345817)
Supplement: Attachment — Submitted filename: Response_to_Reviewers_auresp_2.docx [file pone.0345817.s003.docx]

**Journal Requirements:**

If the reviewer comments include a recommendation to cite specific previously published works, please review and evaluate these publications to determine whether they are relevant and should be cited. There is no requirement to cite these works unless the editor has indicated otherwise. 

Please review your reference list to ensure that it is complete and correct. If you have cited papers that have been retracted, please include the rationale for doing so in the manuscript text, or remove these references and replace them with relevant current references. Any changes to the reference list should be mentioned in the rebuttal letter that accompanies your revised manuscript. If you need to cite a retracted article, indicate the article’s retracted status in the References list and also include a citation and full reference for the retraction notice.


[Note: HTML markup is below. Please do not edit.]

Reviewers' comments:

Reviewer's Responses to Questions

**Comments to the Author**

1. If the authors have adequately addressed your comments raised in a previous round of review and you feel that this manuscript is now acceptable for publication, you may indicate that here to bypass the “Comments to the Author” section, enter your conflict of interest statement in the “Confidential to Editor” section, and submit your "Accept" recommendation.

Reviewer #2: All comments have been addressed

Reviewer #3: (No Response)

2. Is the manuscript technically sound, and do the data support the conclusions?

The manuscript must describe a technically sound piece of scientific research with data that supports the conclusions. Experiments must have been conducted rigorously, with appropriate controls, replication, and sample sizes. The conclusions must be drawn appropriately based on the data presented.

Reviewer #2: Yes

Reviewer #3: (No Response)

3. Has the statistical analysis been performed appropriately and rigorously?

Reviewer #2: Yes

Reviewer #3: Yes

4. Have the authors made all data underlying the findings in their manuscript fully available?

The [PLOS Data policy](http://track.editorialmanager.com/CL0/http:%2F%2Fwww.plosone.org%2Fstatic%2Fpolicies.action%23sharing/1/010f019d22b55194-fd183ddb-06f2-473b-b37b-4cbeeab92a90-000000/MAog5qdjPrudCEOYnqokjgNDTE8RpQHg4jWeVvcTpdk=256) requires authors to make all data underlying the findings described in their manuscript fully available without restriction, with rare exception (please refer to the Data Availability Statement in the manuscript PDF file). The data should be provided as part of the manuscript or its supporting information, or deposited to a public repository. For example, in addition to summary statistics, the data points behind means, medians and variance measures should be available. If there are restrictions on publicly sharing data—e.g. participant privacy or use of data from a third party—those must be specified.

Reviewer #2: Yes

Reviewer #3: Yes

5. Is the manuscript presented in an intelligible fashion and written in standard English?

PLOS ONE does not copyedit accepted manuscripts, so the language in submitted articles must be clear, correct, and unambiguous. Any typographical or grammatical errors should be corrected at revision, so please note any specific errors here.

Reviewer #2: Yes

Reviewer #3: (No Response)

6. Review Comments to the Author

Please use the space provided to explain your answers to the questions above. You may also include additional comments for the author, including concerns about dual publication, research ethics, or publication ethics. (Please upload your review as an attachment if it exceeds 20,000 characters)

**Reviewer #2:** The Authors have made laudable efforts to address all the comments they received. I believe the paper can be accepted with the changes that have been made

**Authors’ response:**
The authors would like to thank the reviewer for the time and effort that he/she spent to review our paper, which in turn had been very helpful in improving the paper. We would also thank him/her for recommending the paper for acceptance.

**Reviewer #3:** Thank you very much for the opportunity to review this manuscript. I have some comments to improve the quality of manuscript.

**Authors’ response****:**
The authors express their gratitude to the reviewer for dedicating the time to review our paper. We have addressed all the comments and thoroughly revised the manuscript to improve the wording. In the following section, we have provided our responses to each of the reviewers’ comments.

**1.** In the introduction, the authors stated that both international and internal migration have significant implications for human health and well-being. However, in the same section, the discussion focuses primarily on internal migration, with little or no mention of international migration. It would be helpful to clarify the reasons for this limited focus on internal migration.

**Authors’ response:**
Thank you for making this comment. In the current project, our main objective is to examine the impact of internal migration, specifically, urban to rural and rural to urban migrations, on the utilization of antenatal care (ANC) in Bangladesh. We chose this focus because of the significant differences in socioeconomic conditions and service provisions between urban and rural areas in the country. However, we recognized that the initial few lines might have caused some confusion, and we have revised them for clarification (please see the lines 48–54 in the document titled ‘Revised Manuscript with Track Changes’). Additionally, we included an explanation about our emphasis on how internal migration influences ANC utilization in the same revised document, in the lines 113–122.

2. Authors should explain clearly regarding the quality of ANC. Authors stated that the quality of ANC was defined as receiving at least four ANC visits, with at least one visit from a qualified healthcare provider, along with regular monitoring of weight and blood pressure, conducting necessary blood and urine tests, and providing information on danger signs during pregnancy. I just wonder if respondents received at least four ANC visits, with at least one visit from a qualified healthcare provider, and got information on danger signs during pregnancy, it could be categorized as taken ANC quality. Or if respondents received regular monitoring of weight and blood pressure, conducting necessary blood and urine tests, and providing information on danger signs during pregnancy, then it can be categorized as taken ANC quality. Please explain in detail how authors categorized the quality of ANC variable into taken and not taken.

**Authors’ response:**
Thank you very much for raising this question. We have revised the section titled ‘Outcome variables’ to improve clarity and precision, aligning our writing with the 2022 BDHS report. The term ‘quality ANC,’ as defined by the 2022 BDHS, refers to a specific concept with particular criteria, as detailed in lines 1–3 of section **9.3 Quality of Antenatal Care** on page 129 in the 2022 BDHS**.** In our paper, we also used ‘quality ANC’ to denote the same specific concept with defined criteria, and we have clarified this definition. Please see the lines 170–185 in the document titled ‘Revised Manuscript with Track Changes’.

7. PLOS authors have the option to publish the peer review history of their article ([what does this mean?](https://track.editorialmanager.com/CL0/https:%2F%2Fjournals.plos.org%2Fplosone%2Fs%2Feditorial-and-peer-review-process%23loc-peer-review-history/1/010f019d22b55194-fd183ddb-06f2-473b-b37b-4cbeeab92a90-000000/fupQI-CoDYfXS7qscRfMKQwvDdDB4MXlf5Yqk1zBAYc=256)). If published, this will include your full peer review and any attached files.


If you choose “no”, your identity will remain anonymous but your review may still be made public.


**Do you want your identity to be public for this peer review?** For information about this choice, including consent withdrawal, please see our [Privacy Policy](https://track.editorialmanager.com/CL0/https:%2F%2Fwww.plos.org%2Fprivacy-policy/1/010f019d22b55194-fd183ddb-06f2-473b-b37b-4cbeeab92a90-000000/JgvOBxS7_17kgsMxMq5RhVyLIJc3gRvcGDq4w4vZfnw=256).

Reviewer #2: No

Reviewer #3: No

[NOTE: If reviewer comments were submitted as an attachment file, they will be attached to this email and accessible via the submission site. Please log into your account, locate the manuscript record, and check for the action link "View Attachments". If this link does not appear, there are no attachment files.]

To ensure your figures meet our technical requirements, please review our figure guidelines: <https://journals.plos.org/plosone/s/figures>

You may also use PLOS’s free figure tool, NAAS, to help you prepare publication quality figures: <https://journals.plos.org/plosone/s/figures#loc-tools-for-figure-preparation>.

NAAS will assess whether your figures meet our technical requirements by comparing each figure against our figure specifications.

*In compliance with data protection regulations, you may request that we remove your personal registration details at any time.* [*(Remove my information/details)*](https://track.editorialmanager.com/CL0/https:%2F%2Fwww.editorialmanager.com%2Fpone%2Flogin.asp%3Fa=r/1/010f019d22b55194-fd183ddb-06f2-473b-b37b-4cbeeab92a90-000000/boFIFTyQWf3jTTesm6O-DlKKonVVHnx_tJnHkqb1jAk=256)*. Please contact the publication office if you have any questions.*
