# Supplementary material for: Influence of internal migration on antenatal care utilization in Bangladesh: Findings from a nationally representative cross-sectional survey
Source: PLoS One. 2026 Jun 26;21(6):e0345817. doi: 10.1371/journal.pone.0345817 (PMC13308873; doi:10.1371/journal.pone.0345817)
Supplement: Attachment — Submitted filename: Response_to_Reviewers_auresp_3.docx [file pone.0345817.s004.docx]

PONE-D-25-39205R2

Influence of internal migration on antenatal care utilization in Bangladesh: Findings from a nationally representative cross-sectional survey

PLOS One

Dear Dr. Rahman,

Thank you for submitting your manuscript to PLOS ONE. After careful consideration, we feel that it has merit but does not fully meet PLOS ONE’s publication criteria as it currently stands. Therefore, we invite you to submit a revised version of the manuscript that addresses the points raised during the review process.

In particular we ask you to address the following minor points that have been raised by a reviewer:

**Authors’ overall response**

The authors thank the academic editor for investing his time and effort in completing the review so quickly. We found all the comments helpful in improving our manuscript, and we have considered all the suggestions.

**- At the outset, pleas**e state how common internal migration in Bangladesh is for the women.

**Authors’ response**

We have added the statistics to the relevant section. Please see lines 70–72 in the file titled Revised Manuscript with Track Changes.

**- Line 97- Please state the** directionality of the accessibility and equity. Was it positive?

**Authors’ response**

We have added an explanation of the directionality. Please see lines 97–103 in the file Revised Manuscript with Track Changes.

**- Line 111-118 - please** remove the example. You could just state that international migration is very uncommon and therefore not considered in this paper.

**Authors’ response**

We have revised the lines as suggested. Please see lines 116–126 in the file Revised Manuscript with Track Changes.

**- Please change the wording** of Line 444. I think we would want to learn from the urban health care settings and strengthen the rural health care structure to improve ANC and other services

**Authors’ response**

We have revised the line as suggested. Please see lines 452–458 in the file Revised Manuscript with Track Changes.

Please submit your revised manuscript by Jul 23 2026 11:59PM. If you will need more time than this to complete your revisions, please reply to this message or contact the journal office at [plosone@plos.org](mailto:plosone@plos.org). When you're ready to submit your revision, log on to <https://www.editorialmanager.com/pone/> and select the 'Submissions Needing Revision' folder to locate your manuscript file.

Please include the following items when submitting your revised manuscript:

- A letter that responds to each point raised by the academic editor and reviewer(s). You should upload this letter as a separate file labeled 'Response to Reviewers'.
- A marked-up copy of your manuscript that highlights changes made to the original version. You should upload this as a separate file labeled 'Revised Manuscript with Track Changes'.
- An unmarked version of your revised paper without tracked changes. You should upload this as a separate file labeled 'Manuscript'.

If you would like to make changes to your financial disclosure, please include your updated statement in your cover letter. Guidelines for resubmitting your figure files are available below the reviewer comments at the end of this letter.

If applicable, we recommend that you deposit your laboratory protocols in [protocols.io](http://protocols.io/) to enhance the reproducibility of your results. Protocols.io assigns your protocol its own identifier (DOI) so that it can be cited independently in the future. For instructions see: [https://journals.plos.org/plosone/s/submission-guidelines#loc-laboratory-protocols](https://track.editorialmanager.com/CL0/https:%2F%2Fjournals.plos.org%2Fplosone%2Fs%2Fsubmission-guidelines%23loc-laboratory-protocols/1/010f019ea9e7036e-d8ad02dd-edce-4df4-b7f9-0e1909975c74-000000/DrYomfqG7d2Lb75LWCMH-Uv0aJlHR8Jlu3wZwTDiYQA=258). Additionally, PLOS ONE offers an option for publishing peer-reviewed Lab Protocol articles, which describe protocols hosted on [protocols.io](http://protocols.io/). Read more information on sharing protocols at [https://plos.org/protocols?utm_medium=editorial-email&utm_source=authorletters&utm_campaign=protocols](https://track.editorialmanager.com/CL0/https:%2F%2Fplos.org%2Fprotocols%3Futm_medium=editorial-email%26utm_source=authorletters%26utm_campaign=protocols/1/010f019ea9e7036e-d8ad02dd-edce-4df4-b7f9-0e1909975c74-000000/C5AmJdZW5jbIX5tLQ1xzMSeuro4_W214tvSafdzRyYI=258).

As the corresponding author, your ORCID iD is verified in the submission system and will appear in the published article. PLOS supports the use of ORCID, and we encourage all coauthors to register for an ORCID iD and use it as well. Please encourage your coauthors to verify their ORCID iD within the submission system before final acceptance, as unverified ORCID iDs will not appear in the published article. *Only* the individual author can complete the verification step; PLOS staff *cannot* verify ORCID iDs on behalf of authors.

We look forward to receiving your revised manuscript.

Kind regards,

Henk Schallig, Ph.D
Academic Editor
PLOS One

Journal Requirements:

1. If the reviewer comments include a recommendation to cite specific previously published works, please review and evaluate these publications to determine whether they are relevant and should be cited. There is no requirement to cite these works unless the editor has indicated otherwise. 

2. Please review your reference list to ensure that it is complete and correct. If you have cited papers that have been retracted, please include the rationale for doing so in the manuscript text, or remove these references and replace them with relevant current references. Any changes to the reference list should be mentioned in the rebuttal letter that accompanies your revised manuscript. If you need to cite a retracted article, indicate the article’s retracted status in the References list and also include a citation and full reference for the retraction notice.

[Note: HTML markup is below. Please do not edit.]

Reviewers' comments:

Reviewer's Responses to Questions

**Comments to the Author**

1. If the authors have adequately addressed your comments raised in a previous round of review and you feel that this manuscript is now acceptable for publication, you may indicate that here to bypass the “Comments to the Author” section, enter your conflict of interest statement in the “Confidential to Editor” section, and submit your "Accept" recommendation.

Reviewer #2: All comments have been addressed

Reviewer #3: All comments have been addressed

2. Is the manuscript technically sound, and do the data support the conclusions?

The manuscript must describe a technically sound piece of scientific research with data that supports the conclusions. Experiments must have been conducted rigorously, with appropriate controls, replication, and sample sizes. The conclusions must be drawn appropriately based on the data presented.

Reviewer #2: Yes

Reviewer #3: Yes

3. Has the statistical analysis been performed appropriately and rigorously?

Reviewer #2: Yes

Reviewer #3: Yes

4. Have the authors made all data underlying the findings in their manuscript fully available?

The [PLOS Data policy](http://track.editorialmanager.com/CL0/http:%2F%2Fwww.plosone.org%2Fstatic%2Fpolicies.action%23sharing/1/010f019ea9e7036e-d8ad02dd-edce-4df4-b7f9-0e1909975c74-000000/bxqajONHTavWz6jBNKyTtjSFJcHJMDLkOt8Jm49Efoc=258) requires authors to make all data underlying the findings described in their manuscript fully available without restriction, with rare exception (please refer to the Data Availability Statement in the manuscript PDF file). The data should be provided as part of the manuscript or its supporting information, or deposited to a public repository. For example, in addition to summary statistics, the data points behind means, medians and variance measures should be available. If there are restrictions on publicly sharing data—e.g. participant privacy or use of data from a third party—those must be specified.

Reviewer #2: Yes

Reviewer #3: Yes

5. Is the manuscript presented in an intelligible fashion and written in standard English?

PLOS ONE does not copyedit accepted manuscripts, so the language in submitted articles must be clear, correct, and unambiguous. Any typographical or grammatical errors should be corrected at revision, so please note any specific errors here.

Reviewer #2: Yes

Reviewer #3: Yes

6. Review Comments to the Author

Please use the space provided to explain your answers to the questions above. You may also include additional comments for the author, including concerns about dual publication, research ethics, or publication ethics. (Please upload your review as an attachment if it exceeds 20,000 characters)

**Reviewer #2:** The authors have strengthened the different sections of the paper. I have a few minor suggestions for consideration.

**Authors’ overall response**

The authors would like to thank the reviewer for the time and effort the reviewer invested to review this paper. We found all the suggestions are likely to improve our manuscript, therefore we have revised the manuscript as per suggestion of the reviewer.

- **At the outset, it ay** be good to state how common is internal migration in Bangladesh for the women.

**Authors’ response**

We have added the statistics to the relevant section. Please see lines 70–72 in the file titled Revised Manuscript with Track Changes.

- **Line 97- Please state the** directionality of the accessibility and equity. Was it positive?

**Authors’ response**

We have added an explanation of the directionality. Please see lines 97–103 in the file Revised Manuscript with Track Changes.

- **Line 111-118 - please** remove the example. You could just state that international migration is very uncommon and therefore not considered in this paper.

**Authors’ response**

We have revised the lines as suggested. Please see lines 116–126 in the file Revised Manuscript with Track Changes.

- **Please change the wording** of Line 444. I think we would want to learn from the urban health care settings and strengthen the rural health care structure to improve ANC and other services

**Authors’ response**

We have revised the line as suggested. Please see lines 452–458 in the file Revised Manuscript with Track Changes.

**Reviewer #3:** The topic of this article is relevant and important. Authors have already addressed all reviewer comments

**Authors’ response**

Thank you very much for your time and consideration.

7. PLOS authors have the option to publish the peer review history of their article ([what does this mean?](https://track.editorialmanager.com/CL0/https:%2F%2Fjournals.plos.org%2Fplosone%2Fs%2Feditorial-and-peer-review-process%23loc-peer-review-history/1/010f019ea9e7036e-d8ad02dd-edce-4df4-b7f9-0e1909975c74-000000/X7uzYXqD_NV2hYDoCVswiOvSVuWgnnhKJL5Bcs-O4Mw=258)). If published, this will include your full peer review and any attached files.


If you choose “no”, your identity will remain anonymous but your review may still be made public.


**Do you want your identity to be public for this peer review?** For information about this choice, including consent withdrawal, please see our [Privacy Policy](https://track.editorialmanager.com/CL0/https:%2F%2Fwww.plos.org%2Fprivacy-policy/1/010f019ea9e7036e-d8ad02dd-edce-4df4-b7f9-0e1909975c74-000000/NZkqGj4UMBnlaUUcm-5gOs1y1QChmjisyv2DdWritYc=258).

Reviewer #2: No

Reviewer #3: No
