# Supplementary material for: Influence of internal migration on antenatal care utilization in Bangladesh: Findings from a nationally representative cross-sectional survey
Source: PLoS One. 2026 Jun 26;21(6):e0345817. doi: 10.1371/journal.pone.0345817 (PMC13308873; doi:10.1371/journal.pone.0345817)
Supplement: Peer review history

# Peer review: Decision Letter 0

**DOI:** 10.1371/journal.pone.0345817.r001

**Authors:** Rumana Rois

5 Oct 2025

PONE-D-25-39205

Association between internal migration and antenatal care in Bangladesh: Evidence from a nationally representative survey

PLOS ONE

Dear Dr. Rahman,

Thank you for submitting your manuscript to PLOS ONE. After careful consideration, we feel that it has merit but does not fully meet PLOS ONE’s publication criteria as it currently stands. Therefore, we invite you to submit a revised version of the manuscript that addresses the points raised during the review process.

Please submit your revised manuscript by Nov 19 2025 11:59PM. If you will need more time than this to complete your revisions, please reply to this message or contact the journal office at plosone@plos.org. When you're ready to submit your revision, log on to https://www.editorialmanager.com/pone/ and select the 'Submissions Needing Revision' folder to locate your manuscript file.

Please include the following items when submitting your revised manuscript:

- A rebuttal letter that responds to each point raised by the academic editor and reviewer(s). You should upload this letter as a separate file labeled 'Response to Reviewers'.
- A marked-up copy of your manuscript that highlights changes made to the original version. You should upload this as a separate file labeled 'Revised Manuscript with Track Changes'.
- An unmarked version of your revised paper without tracked changes. You should upload this as a separate file labeled 'Manuscript'.

If you would like to make changes to your financial disclosure, please include your updated statement in your cover letter. Guidelines for resubmitting your figure files are available below the reviewer comments at the end of this letter.

If applicable, we recommend that you deposit your laboratory protocols in protocols.io to enhance the reproducibility of your results. Protocols.io assigns your protocol its own identifier (DOI) so that it can be cited independently in the future. For instructions see: https://journals.plos.org/plosone/s/submission-guidelines#loc-laboratory-protocols. Additionally, PLOS ONE offers an option for publishing peer-reviewed Lab Protocol articles, which describe protocols hosted on protocols.io. Read more information on sharing protocols at https://plos.org/protocols?utm_medium=editorial-email&utm_source=authorletters&utm_campaign=protocols.

We look forward to receiving your revised manuscript.

Kind regards,

Rumana Rois, PhD

Academic Editor

PLOS ONE

Journal Requirements:

When submitting your revision, we need you to address these additional requirements.

1. Please ensure that your manuscript meets PLOS ONE's style requirements, including those for file naming. The PLOS ONE style templates can be found at https://journals.plos.org/plosone/s/file?id=wjVg/PLOSOne_formatting_sample_main_body.pdf and https://journals.plos.org/plosone/s/file?id=ba62/PLOSOne_formatting_sample_title_authors_affiliations.pdf

2. Your ethics statement should only appear in the Methods section of your manuscript. If your ethics statement is written in any section besides the Methods, please delete it from any other section.

3. If the reviewer comments include a recommendation to cite specific previously published works, please review and evaluate these publications to determine whether they are relevant and should be cited. There is no requirement to cite these works unless the editor has indicated otherwise.

Additional Editor Comments :

Suggestions for improvement:

1. The introduction could be bolstered by incorporating more recent global literature on migration and maternal healthcare, including studies from other South Asian countries.

2. The authors should discuss potential confounders that may not have been fully controlled, including migration duration and reasons for migration.

3. The study could be strengthened by exploring interaction effects, for example, between migration status and wealth or education level.

4. More visual representations of data, such as maps indicating migration flows and their relation to ANC coverage, may enhance reader understanding.

5. Provide more detailed discussion on potential mechanisms behind the observed differences in ANC utilization beyond infrastructure availability, particularly focusing on social and cultural barriers that migrant women might face.

6. Consider expanding the limitations section to address potential recall bias in survey responses and the cross-sectional design's restricted ability to infer causality.

7. The conclusion could be more forward-looking, suggesting areas for future research on maternal health and migration.

[Note: HTML markup is below. Please do not edit.]

Reviewers' comments:

Reviewer's Responses to Questions

**Comments to the Author**

1. Is the manuscript technically sound, and do the data support the conclusions?

The manuscript must describe a technically sound piece of scientific research with data that supports the conclusions. Experiments must have been conducted rigorously, with appropriate controls, replication, and sample sizes. The conclusions must be drawn appropriately based on the data presented.

Reviewer #1: Yes

Reviewer #2: Yes

**********

2. Has the statistical analysis been performed appropriately and rigorously?

Reviewer #1: Yes

Reviewer #2: Yes

**********

3. Have the authors made all data underlying the findings in their manuscript fully available?

The PLOS Data policy requires authors to make all data underlying the findings described in their manuscript fully available without restriction, with rare exception (please refer to the Data Availability Statement in the manuscript PDF file). The data should be provided as part of the manuscript or its supporting information, or deposited to a public repository. For example, in addition to summary statistics, the data points behind means, medians and variance measures should be available. If there are restrictions on publicly sharing data—e.g. participant privacy or use of data from a third party—those must be specified.

Reviewer #1: Yes

Reviewer #2: Yes

**********

4. Is the manuscript presented in an intelligible fashion and written in standard English?

PLOS ONE does not copyedit accepted manuscripts, so the language in submitted articles must be clear, correct, and unambiguous. Any typographical or grammatical errors should be corrected at revision, so please note any specific errors here.

Reviewer #1: No

Reviewer #2: Yes

**********

5. Review Comments to the Author

Please use the space provided to explain your answers to the questions above. You may also include additional comments for the author, including concerns about dual publication, research ethics, or publication ethics. (Please upload your review as an attachment if it exceeds 20,000 characters)

Reviewer #1: Thank you for the opportunity to review this paper. The idea of the paper is good but more work needs to go into this paper. My suggestions are as follows:

1. The authors should revise the abstract and title for clarity, structure, and accuracy (focusing better on the methodology they used to develop findings in the research work)

2. The "Introduction" should be developed, modified, rearranged and divided into some paragraphs based on scientific writing. To strengthen the paper I would suggest consideration to expand the introduction and bring more from literature about the idea of this paper (health equity & healthcare disparities) and shortening the paragraphs; that would make a stronger contribution to the international audiences.

3- The authors should explain more about the type of research (quantitative or Qualitative or Mix Method), in the beginning of "material and method"

4- the authors should mention and justify why they have used the type of statistic test, and mention the statistical software they have employed in this study.

5. The method of extracting and analyzing data from interviews and also validity and reliability of tools is not mentioned.

6. The authors should mention the questionnaire they used for interview.

7. The "result" should be structured based on the main goal (question) & specific goals of the study.

8. Tables are too long and can be divided into 2 or 3 tables to be better for conveying the results of the research.

9. In 'Discussion" the authors should mention the situation of rural healthcare for women in countries with similar context to Bangladesh.

Reviewer #2: The paper addresses an important topic of ANC care and Quality ANC care and the service delivery options in rural and urban settings. I hope that authors can comment on whether migrants more urban to rural settings are worse off than a rural resident. There could be a little more discussion on whether the differences we observe are just because of the service quality and access in rural vs urban settings or do the migrants have a specific experience. The program implications could be strengthened

**********

6. PLOS authors have the option to publish the peer review history of their article (what does this mean?). If published, this will include your full peer review and any attached files.

If you choose “no”, your identity will remain anonymous but your review may still be made public.

**Do you want your identity to be public for this peer review?**  For information about this choice, including consent withdrawal, please see our Privacy Policy.

Reviewer #1: No

Reviewer #2: No

**********

[NOTE: If reviewer comments were submitted as an attachment file, they will be attached to this email and accessible via the submission site. Please log into your account, locate the manuscript record, and check for the action link "View Attachments". If this link does not appear, there are no attachment files.]

While revising your submission, please upload your figure files to the Preflight Analysis and Conversion Engine (PACE) digital diagnostic tool, https://pacev2.apexcovantage.com/. PACE helps ensure that figures meet PLOS requirements. To use PACE, you must first register as a user. Registration is free. Then, login and navigate to the UPLOAD tab, where you will find detailed instructions on how to use the tool. If you encounter any issues or have any questions when using PACE, please email PLOS at figures@plos.org. Please note that Supporting Information files do not need this step.

# Author comment: Author response to Decision Letter 1

**DOI:** 10.1371/journal.pone.0345817.r002

18 Nov 2025

Journal Requirements

When submitting your revision, we need you to address these additional requirements.

1. Please ensure that your manuscript meets PLOS ONE's style requirements, including those for file naming. The PLOS ONE style templates can be found at https://journals.plos.org/plosone/s/file?id=wjVg/PLOSOne_formatting_sample_main_body.pdf and https://journals.plos.org/plosone/s/file?id=ba62/PLOSOne_formatting_sample_title_authors_affiliations.pdf

Authors’ response

Manuscript have been formatted as per PLOS ONE’s guidelines.

2. Your ethics statement should only appear in the Methods section of your manuscript. If your ethics statement is written in any section besides the Methods, please delete it from any other section.

Authors’ response

This guideline has been followed.

3. If the reviewer comments include a recommendation to cite specific previously published works, please review and evaluate these publications to determine whether they are relevant and should be cited. There is no requirement to cite these works unless the editor has indicated otherwise.

Authors’ response

We have followed this guideline.

Additional Editor Comments

Authors’ overall response

The authors would like to thank the editor and reviewers for providing insightful suggestions. The authors consider all the suggestions are very helpful in improving the explanation and readability of the manuscript. Therefore, the authors have addressed all the reasonable suggestions. Besides these, the authors have revised whole manuscript again to improve the sentences and explanations in the manuscript. We appreciate the time and considerations of the editor and reviewers.

Suggestions for improvement:

1. The introduction could be bolstered by incorporating more recent global literature on migration and maternal healthcare, including studies from other South Asian countries.

Authors’ response

Thank you for this insightful comment. We have improved our introduction section as per your suggestion. Please see the lines in the file “Revised Manuscript with Track Changes”: 110-122.

2. The authors should discuss potential confounders that may not have been fully controlled, including migration duration and reasons for migration.

Authors’ response

Thank you for raising this important point. Our main focus was to examine the influence of migration status on ANC utilization. Migration status is the counterpart of migration duration and reasons of migration. Migration status, migration duration, and migration reasons have one category (visitors) in common. Therefore, these variables are very similar in terms of their characteristics. In case of reasons of migration, about 63% of the total selected cases (4306) migrated for marrying a man living in another area, 22% of the total selected cases were non-migrant, 3.4% (or 119 cases) of the total cases migrated for employment, 0.4% (or 17 cases) of the total cases migrated for education, etc. These statistics clearly show that reasons of migration will create a problem of insufficient cell frequency that shall make the drawing of statistical conclusion very difficult. These were some reasons of excluding these variables from the analysis. Nevertheless, based on your suggestion, we added explanatory text in the limitations section noting that these variables may act as unmeasured confounders.

Please see the lines in the file “Revised Manuscript with Track Changes”: 577-580.

3. The study could be strengthened by exploring interaction effects, for example, between migration status and wealth or education level.

Authors’ response

Thank you for this useful suggestion. We explored interaction effects between migration status and both wealth and education. However, the results of the analyses were not meaningful or interpretable. We did not include the interaction effects for the following reasons.

The addition of the interaction effects between migration and education spoilt the model, and all of the interaction effects and migration stream-categories became statistically non-significant in the model. See the Table 1 presenting only the adjusted odds ratios (AOR) of migration and interactions (AORs of other variables have not been presented to make the table concise)

Table 1. Interaction Effects of Migration Stream and Educational Attainment on Utilization of 4+ ANC Visits and Quality ANC Services

Variables Adjusted Odds Ratio of 4* professional ANC (95% CI) Adjusted Odds Ratio of Quality ANC (95% CI)

Main effects

Migrant status (Ref: Urban resident)

Rural resident 0.43 (0.11–1.61) 0.30 (0.04–2.01)

Urban–rural migrant 0.88 (0.33–2.35) 0.70 (0.19–2.55)

Rural–urban migrant 0.65 (0.14–2.97) 0.67 (0.09–4.75)

Visitor 0.71 (0.08–5.95) 1.64 (0.16–17.28)

Education (Ref: No education)

Incomplete primary 0.60 (0.23–1.55) 0.69 (0.20–2.34)

Complete primary 0.87 (0.31–2.42) 0.92 (0.24–3.44)

Incomplete secondary 1.37 (0.61–3.07) 0.95 (0.30–2.97)

Complete secondary/higher 2.19 (0.94–5.09) 1.78 (0.56–5.65)

Interaction effects

Rural × incomplete primary 1.94 (0.40–9.47) 1.73 (0.18–16.36)

Rural × complete primary 2.40 (0.49–11.74) 2.74 (0.32–23.80)

Rural × incomplete secondary 1.87 (0.49–7.20) 3.64 (0.54–24.80)

Rural × complete secondary/higher 1.56 (0.39–6.25) 1.60 (0.23–11.15)

U–R × incomplete primary 1.23 (0.37–4.10) 1.18 (0.27–5.20)

U–R × complete primary 1.32 (0.39–4.45) 1.15 (0.25–5.21)

U–R × incomplete secondary 0.83 (0.30–2.25) 1.31 (0.35–4.90)

U–R × complete secondary/higher 0.68 (0.24–1.92) 0.91 (0.24–3.42)

R–U × incomplete primary 1.74 (0.19–15.85) 0.56 (0.04–8.69)

R–U × complete primary 3.38 (0.46–25.07) 0.93 (0.07–12.57)

R–U × incomplete secondary 2.19 (0.48–10.09) 2.46 (0.30–20.15)

R–U × complete secondary/higher 3.02 (0.56–16.40) 1.69 (0.23–12.72)

Visitor × incomplete primary 2.22 (0.19–25.74) 0.56 (0.03–10.28)

Visitor × complete primary 1.56 (0.14–17.91) 1.10 (0.08–15.89)

Visitor × incomplete secondary 1.26 (0.14–11.29) 0.66 (0.06–7.65)

Visitor × complete secondary/higher 1.51 (0.17–13.13) 0.60 (0.06–6.47)

When the interaction effects of migration and wealth index were introduced in the model that also produced the inconsistent and meaningless results. Relevant results of this model have been presented in Table 2 (AORs of other variables have not been presented to make the table concise). For example, introduction of the interaction effects to the model unusually increased the odds ratio of receiving 4+ professional ANC for urban-rural migrants to 24.12. Besides these, the AORs of all the interaction terms became less than one (1), which is logically inconsistent. For example, when rural people moved to urban areas, they were more likely to have 4+ professional ANC than the urban residents, that is their AOR was >1. In the same way, if a richest rural person moved to urban area, his odds of receiving 4+ professional ANC should have been more than one or more than those who were poor urban residents. But Table 2 shows that the odds of receiving 4+ professional ANC by the richest rural persons who moved to urban areas (AOR: 0.07, 95% CI: 0.01–0.61, p<0.05) was less than those who were poor urban residents. Most of the AORs presented in the Table 2 could be logically inconsistent in the same way.

Table 2: Interaction Effects of Migration Stream and wealth index on Utilization of 4+ ANC Visits and Quality ANC Services

Variable Adjusted Odds Ratio of ≥4 professional ANC (95% CI) Adjusted Odds Ratio of Quality ANC (95% CI)

Main effects

Migrant status (Ref: Urban resident) 1.30 (0.60–2.82) 3.08 (0.91–10.38)

Rural resident 1.38 (0.72–2.64) 2.99 (1.05–8.51)**

Urban–rural migrant 24.12 (3.05–191.01)*** 4.16 (0.41–42.64)

Rural–urban migrant 2.65 (1.01–6.97)** 4.26 (1.05–17.38)**

Wealth Index (ref: poorest)

Poorer 2.43 (1.27–4.64)*** 6.44 (1.85–22.50)***

Middle 1.89 (0.90–3.97) 5.23 (1.78–15.39)***

Richer 2.90 (1.54–5.46)*** 7.63 (2.65–21.95)***

Richest 6.06 (3.05–12.05)*** 11.31 (3.89–32.89)***

Interaction: Migration Stream × Wealth Status (ref: Non-migrant × Poor)

Rural Resident × Poor 0.51 (0.21–1.23) 0.20 (0.05–0.88)**

Rural Resident × Middle 0.80 (0.31–2.08) 0.27 (0.07–0.98)**

Rural Resident × Richer 0.64 (0.26–1.57) 0.22 (0.06–0.82)**

Rural Resident × Richest 0.32 (0.13–0.83)** 0.16 (0.04–0.59)***

U-R × Poor 0.43 (0.21–0.87)** 0.17 (0.05–0.63)***

U-R × Middle 0.65 (0.29–1.46) 0.29 (0.09–0.91)**

U-R × Richer 0.57 (0.28–1.16) 0.26 (0.09–0.80)**

U-R × Richest 0.37 (0.17–0.80)** 0.24 (0.08–0.75)**

R-U × Poor 0.20 (0.01–2.80) 0.39 (0.02–6.21)

R-U × Middle 0.04 (0.00–0.47)*** 0.38 (0.03–5.71)

R-U × Richer 0.05 (0.01–0.48)*** 0.22 (0.02–2.64)

R-U × Richest 0.07 (0.01–0.61)** 0.28 (0.03–3.08)

Visitors × Poor 0.29 (0.10–0.84)** 0.19 (0.04–1.03)

Visitors × Middle 0.90 (0.30–2.73) 0.38 (0.09–1.63)

Visitors × Richer 1.34 (0.48–3.77) 0.50 (0.12–2.04)

Visitors × Richest 0.85 (0.30–2.45) 0.40 (0.09–1.72)

Note: Both models are adjusted for migration duration, migration cause age group, age at first marriage, birth order, media exposure, education, work status, religion, and wealth quintile. Reference category is denoted by (ref). Significance: ***p < 0.01, **p < 0.05.

4. More visual representations of data, such as maps indicating migration flows and their relation to ANC coverage, may enhance reader understanding.

Authors’ response

Thank you for the suggestion. The BDHS does not provide necessary geographic location indicating individual’s migration from one type of area to other types; therefore, we could not make map to show the migration flows and the changes in the ANC use rates. Nevertheless, we have made flow charts to make it is easier to understand the migration stream and the differences in the odds of receiving ANC services. Please see the figures 3 and 4 in the file “Revised Manuscript with Track Changes”.

5. Provide more detailed discussion on potential mechanisms behind the observed differences in ANC utilization beyond infrastructure availability, particularly focusing on social and cultural barriers that migrant women might face.

Authors’ response

Thank you for this important suggestion. We expanded the Discussion to include sociocultural and psychosocial mechanisms that affect migrant women’s healthcare-seeking behaviour. Please see the lines in “Revised Manuscript with Track Changes”: 507-514.

6. Consider expanding the limitations section to address potential recall bias in survey responses and the cross-sectional design's restricted ability to infer causality.

Authors’ response

We added a detailed paragraph addressing these concerns. Please see the lines in “Revised Manuscript with Track Changes”: 573-577.

7. The conclusion could be more forward-looking, suggesting areas for future research on maternal health and migration.

Authors’ response

We revised the Conclusion to include directions for future research. Please see following lines in “Revised Manuscript with Track Changes”: 599-606.

--------------------------------------------------------------------------------------------------------------------------

[Note: HTML markup is below. Please do not edit.]

Reviewers' comments:

Reviewer's Responses to Questions

Comments to the Author

1. Is the manuscript technically sound, and do the data support the conclusions?

The manuscript must describe a technically sound piece of scientific research with data that supports the conclusions. Experiments must have been conducted rigorously, with appropriate controls, replication, and sample sizes. The conclusions must be drawn appropriately based on the data presented.

Reviewer #1: Yes

Reviewer #2: Yes

________________________________________

2. Has the statistical analysis been performed appropriately and rigorously?

Reviewer #1: Yes

Reviewer #2: Yes

________________________________________

3. Have the authors made all data underlying the findings in their manuscript fully available?

The PLOS Data policy requires authors to make all data underlying the findings described in their manuscript fully available without restriction, with rare exception (please refer to the Data Availability Statement in the manuscript PDF file). The data should be provided as part of the manuscript or its supporting information, or deposited to a public repository. For example, in addition to summary statistics, the data points behind means, medians and variance measures should be available. If there are restrictions on publicly sharing data—e.g. participant privacy or use of data from a third party—those must be specified.

Reviewer #1: Yes

Reviewer #2: Yes

________________________________________

4. Is the manuscript presented in an intelligible fashion and written in standard English?

PLOS ONE does not copyedit accepted manuscripts, so the language in submitted articles must be clear, correct, and unambiguous. Any typographical or grammatical errors should be corrected at revision, so please note any specific errors here.

Reviewer #1: No

Reviewer #2: Yes

________________________________________

5. Review Comments to the Author

Please use the space provided to explain your answers to the questions above. You may also include additional comments for the author, including concerns about dual publication, research ethics, or publication ethics. (Please upload your review as an attachment if it exceeds 20,000 characters)

Reviewer #1

Thank you for the opportunity to review this paper. The idea of the paper is good but more work needs to go into this paper. My suggestions are as follows:

1. The authors should revise the abstract and title for clarity, structure, and accuracy (focusing better on the methodology they used to develop findings in the research work)

Authors’ response

The abstract and title were revised to improve clarity, structure, and methodological transparency. Please see the title and abstract in “Revised Manuscript with Track Changes.”

2. The "Introduction" should be developed, modified, rearranged and divided into some paragraphs based on scientific writing. To strengthen the paper I would suggest consideration to expand the introduction and bring more from literature about the idea of this paper (health equity & healthcare disparities) and shortening the paragraphs; that would make a stronger contribution to the international audiences.

Authors’ response

Thank you for this suggestion. The Introduction was reorganized into multiple focused paragraphs, shortened for clarity, and strengthened with recent global and South Asian evidence on health equity, healthcare disparities, and migration. Please see the introduction’s track changed portions in “Revised Manuscript with Track Changes.”

3- The authors should explain more about the type of research (quantitative or Qualitative or Mix Method), in the beginning of "material and method"

Authors’ response

To address this suggestion, we have added following lines in “Revised Manuscript with Track Changes”: 189.

4- the authors should mention and justify why they have used the type of statistic test, and mention the statistical software they have employed in this study.

Authors’ response

Thank you. We clarified the analytical justification and software: Please see following lines in “Revised Manuscript with Track Changes”: 285-305.

5. The method of extracting and analyzing data from interviews and also validity and reliability of tools is not mentioned.

Authors’ response

We have addressed these suggestions in following lines in “Revised Manuscript with Track Changes.”: 206-211.

6. The authors should mention the questionnaire they used for interview.

Authors’ response

We have provided information about the questionnaire in the lines in “Revised Manuscript with Track Changes.”: 222

7. The "result" should be structured based on the main goal (question) & specific goals of the study.

Authors’ response

We have tried to address this suggestion by breaking the result section into two parts namely Correlates of receiving four or more professional ANC and Correlates of receiving Quality ANC visits. In these sections, we further subdivided the results. For example, as our main focus was on the association between internal migration and ANC utilization, we have presente

Attachment Submitted filename: Response to Reviewers.docx

# Peer review: Decision Letter 1

**DOI:** 10.1371/journal.pone.0345817.r003

**Authors:** Katrien G. Janin

24 Mar 2026

<div>PONE-D-25-39205R1

Influence of internal migration on antenatal care utilization in Bangladesh: Findings from a nationally representative cross-sectional survey

PLOS One

Dear Dr. Rahman,

Thank you for submitting your manuscript to PLOS ONE. After careful consideration, we feel that it has merit but does not fully meet PLOS ONE’s publication criteria as it currently stands. Therefore, we invite you to submit a revised version of the manuscript that addresses the points raised during the review process.

The manuscript has been evaluated by two reviewers, and their comments are provided below. Although they acknowledged the improvements in the revised version, they also identified several remaining concerns. Please address these points carefully when preparing your revised manuscript and your point‑by‑point response.

Please submit your revised manuscript by May 08 2026 11:59PM. If you will need more time than this to complete your revisions, please reply to this message or contact the journal office at plosone@plos.org. When you're ready to submit your revision, log on to https://www.editorialmanager.com/pone/ and select the 'Submissions Needing Revision' folder to locate your manuscript file.

Please include the following items when submitting your revised manuscript:

- A letter that responds to each point raised by the academic editor and reviewer(s). You should upload this letter as a separate file labeled 'Response to Reviewers'.
- A marked-up copy of your manuscript that highlights changes made to the original version. You should upload this as a separate file labeled 'Revised Manuscript with Track Changes'.
- An unmarked version of your revised paper without tracked changes. You should upload this as a separate file labeled 'Manuscript'.

If you would like to make changes to your financial disclosure, please include your updated statement in your cover letter. Guidelines for resubmitting your figure files are available below the reviewer comments at the end of this letter.

If applicable, we recommend that you deposit your laboratory protocols in protocols.io to enhance the reproducibility of your results. Protocols.io assigns your protocol its own identifier (DOI) so that it can be cited independently in the future. For instructions see: https://journals.plos.org/plosone/s/submission-guidelines#loc-laboratory-protocols. Additionally, PLOS ONE offers an option for publishing peer-reviewed Lab Protocol articles, which describe protocols hosted on protocols.io. Read more information on sharing protocols at https://plos.org/protocols?utm_medium=editorial-email&utm_source=authorletters&utm_campaign=protocols.

As the corresponding author, your ORCID iD is verified in the submission system and will appear in the published article. PLOS supports the use of ORCID, and we encourage all coauthors to register for an ORCID iD and use it as well. Please encourage your coauthors to verify their ORCID iD within the submission system before final acceptance, as unverified ORCID iDs will not appear in the published article. *Only*  the individual author can complete the verification step; PLOS staff *cannot*  verify ORCID iDs on behalf of authors.

We look forward to receiving your revised manuscript.

Kind regards,

Katrien G. Janin, PhD

Staff Editor

PLOS One

Journal Requirements:

If the reviewer comments include a recommendation to cite specific previously published works, please review and evaluate these publications to determine whether they are relevant and should be cited. There is no requirement to cite these works unless the editor has indicated otherwise.

Please review your reference list to ensure that it is complete and correct. If you have cited papers that have been retracted, please include the rationale for doing so in the manuscript text, or remove these references and replace them with relevant current references. Any changes to the reference list should be mentioned in the rebuttal letter that accompanies your revised manuscript. If you need to cite a retracted article, indicate the article’s retracted status in the References list and also include a citation and full reference for the retraction notice.

[Note: HTML markup is below. Please do not edit.]

Reviewers' comments:

Reviewer's Responses to Questions

**Comments to the Author**

1. If the authors have adequately addressed your comments raised in a previous round of review and you feel that this manuscript is now acceptable for publication, you may indicate that here to bypass the “Comments to the Author” section, enter your conflict of interest statement in the “Confidential to Editor” section, and submit your "Accept" recommendation.

Reviewer #2: All comments have been addressed

Reviewer #3: (No Response)

**********

2. Is the manuscript technically sound, and do the data support the conclusions?

The manuscript must describe a technically sound piece of scientific research with data that supports the conclusions. Experiments must have been conducted rigorously, with appropriate controls, replication, and sample sizes. The conclusions must be drawn appropriately based on the data presented.

Reviewer #2: Yes

Reviewer #3: (No Response)

**********

3. Has the statistical analysis been performed appropriately and rigorously?

Reviewer #2: Yes

Reviewer #3: Yes

**********

4. Have the authors made all data underlying the findings in their manuscript fully available?

The PLOS Data policy requires authors to make all data underlying the findings described in their manuscript fully available without restriction, with rare exception (please refer to the Data Availability Statement in the manuscript PDF file). The data should be provided as part of the manuscript or its supporting information, or deposited to a public repository. For example, in addition to summary statistics, the data points behind means, medians and variance measures should be available. If there are restrictions on publicly sharing data—e.g. participant privacy or use of data from a third party—those must be specified.

Reviewer #2: Yes

Reviewer #3: Yes

**********

5. Is the manuscript presented in an intelligible fashion and written in standard English?

PLOS ONE does not copyedit accepted manuscripts, so the language in submitted articles must be clear, correct, and unambiguous. Any typographical or grammatical errors should be corrected at revision, so please note any specific errors here.

Reviewer #2: Yes

Reviewer #3: (No Response)

**********

6. Review Comments to the Author

Please use the space provided to explain your answers to the questions above. You may also include additional comments for the author, including concerns about dual publication, research ethics, or publication ethics. (Please upload your review as an attachment if it exceeds 20,000 characters)

Reviewer #2: The Authors have made laudable efforts to address all the comments they received. I believe the paper can be accepted with the changes that have been made

Reviewer #3: Thank you very much for the opportunity to review this manuscript. I have some comments to improve the quality of manuscript

1. In the introduction, the authors stated that both international and internal migration have significant implications for human health and well-being. However, in the same section, the discussion focuses primarily on internal migration, with little or no mention of international migration. It would be helpful to clarify the reasons for this limited focus on internal migration.

2. Authors should explain clearly regarding the quality of ANC. Authors stated that the quality of ANC was defined as receiving at least four ANC visits, with at least one visit from a qualified healthcare provider, along with regular monitoring of weight and blood pressure, conducting necessary blood and urine tests, and providing information on danger signs during pregnancy. I just wonder if respondents received at least four ANC visits, with at least one visit from a qualified healthcare provider, and got information on danger signs during pregnancy, it could be categorized as taken ANC quality. Or if respondents received regular monitoring of weight and blood pressure, conducting necessary blood and urine tests, and providing information on danger signs during pregnancy, then it can be categorized as taken ANC quality. Please explain in detail how authors categorized the quality of ANC variable into taken and not taken

**********

7. PLOS authors have the option to publish the peer review history of their article (what does this mean?). If published, this will include your full peer review and any attached files.

If you choose “no”, your identity will remain anonymous but your review may still be made public.

**Do you want your identity to be public for this peer review?**  For information about this choice, including consent withdrawal, please see our Privacy Policy.

Reviewer #2: No

Reviewer #3: No

**********

[NOTE: If reviewer comments were submitted as an attachment file, they will be attached to this email and accessible via the submission site. Please log into your account, locate the manuscript record, and check for the action link "View Attachments". If this link does not appear, there are no attachment files.]

To ensure your figures meet our technical requirements, please review our figure guidelines: https://journals.plos.org/plosone/s/figures

You may also use PLOS’s free figure tool, NAAS, to help you prepare publication quality figures: https://journals.plos.org/plosone/s/figures#loc-tools-for-figure-preparation.

NAAS will assess whether your figures meet our technical requirements by comparing each figure against our figure specifications.

# Author comment: Author response to Decision Letter 2

**DOI:** 10.1371/journal.pone.0345817.r004

5 Apr 2026

Journal Requirements:

If the reviewer comments include a recommendation to cite specific previously published works, please review and evaluate these publications to determine whether they are relevant and should be cited. There is no requirement to cite these works unless the editor has indicated otherwise.

Please review your reference list to ensure that it is complete and correct. If you have cited papers that have been retracted, please include the rationale for doing so in the manuscript text, or remove these references and replace them with relevant current references. Any changes to the reference list should be mentioned in the rebuttal letter that accompanies your revised manuscript. If you need to cite a retracted article, indicate the article’s retracted status in the References list and also include a citation and full reference for the retraction notice.

[Note: HTML markup is below. Please do not edit.]

Reviewers' comments:

Reviewer's Responses to Questions

Comments to the Author

1. If the authors have adequately addressed your comments raised in a previous round of review and you feel that this manuscript is now acceptable for publication, you may indicate that here to bypass the “Comments to the Author” section, enter your conflict of interest statement in the “Confidential to Editor” section, and submit your "Accept" recommendation.

Reviewer #2: All comments have been addressed

Reviewer #3: (No Response)

________________________________________

2. Is the manuscript technically sound, and do the data support the conclusions?

The manuscript must describe a technically sound piece of scientific research with data that supports the conclusions. Experiments must have been conducted rigorously, with appropriate controls, replication, and sample sizes. The conclusions must be drawn appropriately based on the data presented.

Reviewer #2: Yes

Reviewer #3: (No Response)

________________________________________

3. Has the statistical analysis been performed appropriately and rigorously?

Reviewer #2: Yes

Reviewer #3: Yes

________________________________________

4. Have the authors made all data underlying the findings in their manuscript fully available?

The PLOS Data policy requires authors to make all data underlying the findings described in their manuscript fully available without restriction, with rare exception (please refer to the Data Availability Statement in the manuscript PDF file). The data should be provided as part of the manuscript or its supporting information, or deposited to a public repository. For example, in addition to summary statistics, the data points behind means, medians and variance measures should be available. If there are restrictions on publicly sharing data—e.g. participant privacy or use of data from a third party—those must be specified.

Reviewer #2: Yes

Reviewer #3: Yes

________________________________________

5. Is the manuscript presented in an intelligible fashion and written in standard English?

PLOS ONE does not copyedit accepted manuscripts, so the language in submitted articles must be clear, correct, and unambiguous. Any typographical or grammatical errors should be corrected at revision, so please note any specific errors here.

Reviewer #2: Yes

Reviewer #3: (No Response)

________________________________________

6. Review Comments to the Author

Please use the space provided to explain your answers to the questions above. You may also include additional comments for the author, including concerns about dual publication, research ethics, or publication ethics. (Please upload your review as an attachment if it exceeds 20,000 characters)

Reviewer #2: The Authors have made laudable efforts to address all the comments they received. I believe the paper can be accepted with the changes that have been made

Authors’ response:

The authors would like to thank the reviewer for the time and effort that he/she spent to review our paper, which in turn had been very helpful in improving the paper. We would also thank him/her for recommending the paper for acceptance.

Reviewer #3: Thank you very much for the opportunity to review this manuscript. I have some comments to improve the quality of manuscript.

Authors’ response:

The authors express their gratitude to the reviewer for dedicating the time to review our paper. We have addressed all the comments and thoroughly revised the manuscript to improve the wording. In the following section, we have provided our responses to each of the reviewers’ comments.

1. In the introduction, the authors stated that both international and internal migration have significant implications for human health and well-being. However, in the same section, the discussion focuses primarily on internal migration, with little or no mention of international migration. It would be helpful to clarify the reasons for this limited focus on internal migration.

Authors’ response:

Thank you for making this comment. In the current project, our main objective is to examine the impact of internal migration, specifically, urban to rural and rural to urban migrations, on the utilization of antenatal care (ANC) in Bangladesh. We chose this focus because of the significant differences in socioeconomic conditions and service provisions between urban and rural areas in the country. However, we recognized that the initial few lines might have caused some confusion, and we have revised them for clarification (please see the lines 48–54 in the document titled ‘Revised Manuscript with Track Changes’). Additionally, we included an explanation about our emphasis on how internal migration influences ANC utilization in the same revised document, in the lines 113–122.

2. Authors should explain clearly regarding the quality of ANC. Authors stated that the quality of ANC was defined as receiving at least four ANC visits, with at least one visit from a qualified healthcare provider, along with regular monitoring of weight and blood pressure, conducting necessary blood and urine tests, and providing information on danger signs during pregnancy. I just wonder if respondents received at least four ANC visits, with at least one visit from a qualified healthcare provider, and got information on danger signs during pregnancy, it could be categorized as taken ANC quality. Or if respondents received regular monitoring of weight and blood pressure, conducting necessary blood and urine tests, and providing information on danger signs during pregnancy, then it can be categorized as taken ANC quality. Please explain in detail how authors categorized the quality of ANC variable into taken and not taken.

Authors’ response:

Thank you very much for raising this question. We have revised the section titled ‘Outcome variables’ to improve clarity and precision, aligning our writing with the 2022 BDHS report. The term ‘quality ANC,’ as defined by the 2022 BDHS, refers to a specific concept with particular criteria, as detailed in lines 1–3 of section 9.3 Quality of Antenatal Care on page 129 in the 2022 BDHS. In our paper, we also used ‘quality ANC’ to denote the same specific concept with defined criteria, and we have clarified this definition. Please see the lines 170–185 in the document titled ‘Revised Manuscript with Track Changes’.

________________________________________

7. PLOS authors have the option to publish the peer review history of their article (what does this mean?). If published, this will include your full peer review and any attached files.

If you choose “no”, your identity will remain anonymous but your review may still be made public.

Do you want your identity to be public for this peer review? For information about this choice, including consent withdrawal, please see our Privacy Policy.

Reviewer #2: No

Reviewer #3: No

________________________________________

[NOTE: If reviewer comments were submitted as an attachment file, they will be attached to this email and accessible via the submission site. Please log into your account, locate the manuscript record, and check for the action link "View Attachments". If this link does not appear, there are no attachment files.]

To ensure your figures meet our technical requirements, please review our figure guidelines: https://journals.plos.org/plosone/s/figures

You may also use PLOS’s free figure tool, NAAS, to help you prepare publication quality figures: https://journals.plos.org/plosone/s/figures#loc-tools-for-figure-preparation.

NAAS will assess whether your figures meet our technical requirements by comparing each figure against our figure specifications.

________________________________________

In compliance with data protection regulations, you may request that we remove your personal registration details at any time. (Remove my information/details). Please contact the publication office if you have any questions.

Attachment Submitted filename: Response_to_Reviewers_auresp_2.docx

# Peer review: Decision Letter 2

**DOI:** 10.1371/journal.pone.0345817.r005

**Authors:** Henk Schallig

8 Jun 2026

PONE-D-25-39205R2

Influence of internal migration on antenatal care utilization in Bangladesh: Findings from a nationally representative cross-sectional survey

PLOS One

Dear Dr. Rahman,

Thank you for submitting your manuscript to PLOS ONE. After careful consideration, we feel that it has merit but does not fully meet PLOS ONE’s publication criteria as it currently stands. Therefore, we invite you to submit a revised version of the manuscript that addresses the points raised during the review process.

In particular we ask you to address the following minor points that have beenr aised by a reviewer:

- At the outset, please state how common internal migration in Bangladesh is for the women.

- Line 97- Please state the directionality of the accessibility and equity. Was it positive?

- Line 111-118 - please remove the example. You could just state that international migration is very uncommon and therefore not considered in this paper.

- Please change the wording of Line 444. I think we would want to learn from the urban health care settings and strengthen the rural health care structure to improve ANC and other services

Please submit your revised manuscript by Jul 23 2026 11:59PM. If you will need more time than this to complete your revisions, please reply to this message or contact the journal office at plosone@plos.org. When you're ready to submit your revision, log on to https://www.editorialmanager.com/pone/ and select the 'Submissions Needing Revision' folder to locate your manuscript file.

Please include the following items when submitting your revised manuscript:

- A letter that responds to each point raised by the academic editor and reviewer(s). You should upload this letter as a separate file labeled 'Response to Reviewers'.
- A marked-up copy of your manuscript that highlights changes made to the original version. You should upload this as a separate file labeled 'Revised Manuscript with Track Changes'.
- An unmarked version of your revised paper without tracked changes. You should upload this as a separate file labeled 'Manuscript'.

If you would like to make changes to your financial disclosure, please include your updated statement in your cover letter. Guidelines for resubmitting your figure files are available below the reviewer comments at the end of this letter.

If applicable, we recommend that you deposit your laboratory protocols in protocols.io to enhance the reproducibility of your results. Protocols.io assigns your protocol its own identifier (DOI) so that it can be cited independently in the future. For instructions see: https://journals.plos.org/plosone/s/submission-guidelines#loc-laboratory-protocols. Additionally, PLOS ONE offers an option for publishing peer-reviewed Lab Protocol articles, which describe protocols hosted on protocols.io. Read more information on sharing protocols at https://plos.org/protocols?utm_medium=editorial-email&utm_source=authorletters&utm_campaign=protocols.

As the corresponding author, your ORCID iD is verified in the submission system and will appear in the published article. PLOS supports the use of ORCID, and we encourage all coauthors to register for an ORCID iD and use it as well. Please encourage your coauthors to verify their ORCID iD within the submission system before final acceptance, as unverified ORCID iDs will not appear in the published article. *Only*  the individual author can complete the verification step; PLOS staff *cannot*  verify ORCID iDs on behalf of authors.

We look forward to receiving your revised manuscript.

Kind regards,

Henk Schallig, Ph.D

Academic Editor

PLOS One

Journal Requirements:

1. If the reviewer comments include a recommendation to cite specific previously published works, please review and evaluate these publications to determine whether they are relevant and should be cited. There is no requirement to cite these works unless the editor has indicated otherwise.

2. Please review your reference list to ensure that it is complete and correct. If you have cited papers that have been retracted, please include the rationale for doing so in the manuscript text, or remove these references and replace them with relevant current references. Any changes to the reference list should be mentioned in the rebuttal letter that accompanies your revised manuscript. If you need to cite a retracted article, indicate the article’s retracted status in the References list and also include a citation and full reference for the retraction notice.

[Note: HTML markup is below. Please do not edit.]

Reviewers' comments:

Reviewer's Responses to Questions

**Comments to the Author**

1. If the authors have adequately addressed your comments raised in a previous round of review and you feel that this manuscript is now acceptable for publication, you may indicate that here to bypass the “Comments to the Author” section, enter your conflict of interest statement in the “Confidential to Editor” section, and submit your "Accept" recommendation.

Reviewer #2: All comments have been addressed

Reviewer #3: All comments have been addressed

**********

2. Is the manuscript technically sound, and do the data support the conclusions?

The manuscript must describe a technically sound piece of scientific research with data that supports the conclusions. Experiments must have been conducted rigorously, with appropriate controls, replication, and sample sizes. The conclusions must be drawn appropriately based on the data presented.

Reviewer #2: Yes

Reviewer #3: Yes

**********

3. Has the statistical analysis been performed appropriately and rigorously?

Reviewer #2: Yes

Reviewer #3: Yes

**********

4. Have the authors made all data underlying the findings in their manuscript fully available?

The PLOS Data policy requires authors to make all data underlying the findings described in their manuscript fully available without restriction, with rare exception (please refer to the Data Availability Statement in the manuscript PDF file). The data should be provided as part of the manuscript or its supporting information, or deposited to a public repository. For example, in addition to summary statistics, the data points behind means, medians and variance measures should be available. If there are restrictions on publicly sharing data—e.g. participant privacy or use of data from a third party—those must be specified.

Reviewer #2: Yes

Reviewer #3: Yes

**********

5. Is the manuscript presented in an intelligible fashion and written in standard English?

PLOS ONE does not copyedit accepted manuscripts, so the language in submitted articles must be clear, correct, and unambiguous. Any typographical or grammatical errors should be corrected at revision, so please note any specific errors here.

Reviewer #2: Yes

Reviewer #3: Yes

**********

6. Review Comments to the Author

Please use the space provided to explain your answers to the questions above. You may also include additional comments for the author, including concerns about dual publication, research ethics, or publication ethics. (Please upload your review as an attachment if it exceeds 20,000 characters)

Reviewer #2: The authors have strengthened the different sections of the paper. I have a few minor suggestions for consideration. At the outset, it ay be good to state how common is internal migration in Bangladesh for the women. Line 97- Please state the directionality of the accessibility and equity. Was it positive? Line 111-118 - please remove the example. You could just state that international migration is very uncommon and therefore not considered in this paper. Please change the wording of Line 444. I think we would want to learn from the urban health care settings and strengthen the rural health care structure to improve ANC and other services

Reviewer #3: The topic of this article is relevant and important. Authors have already addressed all reviewer comments

**********

7. PLOS authors have the option to publish the peer review history of their article (what does this mean?). If published, this will include your full peer review and any attached files.

If you choose “no”, your identity will remain anonymous but your review may still be made public.

**Do you want your identity to be public for this peer review?**  For information about this choice, including consent withdrawal, please see our Privacy Policy.

Reviewer #2: No

Reviewer #3: No

**********

[NOTE: If reviewer comments were submitted as an attachment file, they will be attached to this email and accessible via the submission site. Please log into your account, locate the manuscript record, and check for the action link "View Attachments". If this link does not appear, there are no attachment files.]

To ensure your figures meet our technical requirements, please review our figure guidelines: https://journals.plos.org/plosone/s/figures

You may also use PLOS’s free figure tool, NAAS, to help you prepare publication quality figures: https://journals.plos.org/plosone/s/figures#loc-tools-for-figure-preparation.

NAAS will assess whether your figures meet our technical requirements by comparing each figure against our figure specifications.

# Author comment: Author response to Decision Letter 3

**DOI:** 10.1371/journal.pone.0345817.r006

10 Jun 2026

PONE-D-25-39205R2

Influence of internal migration on antenatal care utilization in Bangladesh: Findings from a nationally representative cross-sectional survey

PLOS One

Dear Dr. Rahman,

Thank you for submitting your manuscript to PLOS ONE. After careful consideration, we feel that it has merit but does not fully meet PLOS ONE’s publication criteria as it currently stands. Therefore, we invite you to submit a revised version of the manuscript that addresses the points raised during the review process.

In particular we ask you to address the following minor points that have been raised by a reviewer:

Authors’ overall response

The authors thank to the academic editor for investing his time and effort in completing the review so quickly. We found all the comments helpful in improving our manuscript. Therefore, we have considered all the suggestions.

- At the outset, please state how common internal migration in Bangladesh is for the women.

Authors’ response

We have added the statistics at the relevant place. Please see the lines (70–72) in the file named Revised Manuscript with Track Changes.

- Line 97- Please state the directionality of the accessibility and equity. Was it positive?

Authors’ response

We have added the explanation about the directionality. Please see the lines 97–103 in the file Revised Manuscript with Track Changes.

- Line 111-118 - please remove the example. You could just state that international migration is very uncommon and therefore not considered in this paper.

Authors’ response

We have revised the lines as per suggestion. Please see the lines 116–126 in the file Revised Manuscript with Track Changes.

- Please change the wording of Line 444. I think we would want to learn from the urban health care settings and strengthen the rural health care structure to improve ANC and other services

Authors’ response

We have revised the line as per suggestion. Please see the lines 452–458 in the file Revised Manuscript with Track Changes.

Please submit your revised manuscript by Jul 23 2026 11:59PM. If you will need more time than this to complete your revisions, please reply to this message or contact the journal office at plosone@plos.org. When you're ready to submit your revision, log on to https://www.editorialmanager.com/pone/ and select the 'Submissions Needing Revision' folder to locate your manuscript file.

Please include the following items when submitting your revised manuscript:

• A letter that responds to each point raised by the academic editor and reviewer(s). You should upload this letter as a separate file labeled 'Response to Reviewers'.

• A marked-up copy of your manuscript that highlights changes made to the original version. You should upload this as a separate file labeled 'Revised Manuscript with Track Changes'.

• An unmarked version of your revised paper without tracked changes. You should upload this as a separate file labeled 'Manuscript'.

If you would like to make changes to your financial disclosure, please include your updated statement in your cover letter. Guidelines for resubmitting your figure files are available below the reviewer comments at the end of this letter.

If applicable, we recommend that you deposit your laboratory protocols in protocols.io to enhance the reproducibility of your results. Protocols.io assigns your protocol its own identifier (DOI) so that it can be cited independently in the future. For instructions see: https://journals.plos.org/plosone/s/submission-guidelines#loc-laboratory-protocols. Additionally, PLOS ONE offers an option for publishing peer-reviewed Lab Protocol articles, which describe protocols hosted on protocols.io. Read more information on sharing protocols at https://plos.org/protocols?utm_medium=editorial-email&utm_source=authorletters&utm_campaign=protocols.

As the corresponding author, your ORCID iD is verified in the submission system and will appear in the published article. PLOS supports the use of ORCID, and we encourage all coauthors to register for an ORCID iD and use it as well. Please encourage your coauthors to verify their ORCID iD within the submission system before final acceptance, as unverified ORCID iDs will not appear in the published article. Only the individual author can complete the verification step; PLOS staff cannot verify ORCID iDs on behalf of authors.

We look forward to receiving your revised manuscript.

Kind regards,

Henk Schallig, Ph.D

Academic Editor

PLOS One

Journal Requirements:

1. If the reviewer comments include a recommendation to cite specific previously published works, please review and evaluate these publications to determine whether they are relevant and should be cited. There is no requirement to cite these works unless the editor has indicated otherwise.

2. Please review your reference list to ensure that it is complete and correct. If you have cited papers that have been retracted, please include the rationale for doing so in the manuscript text, or remove these references and replace them with relevant current references. Any changes to the reference list should be mentioned in the rebuttal letter that accompanies your revised manuscript. If you need to cite a retracted article, indicate the article’s retracted status in the References list and also include a citation and full reference for the retraction notice.

[Note: HTML markup is below. Please do not edit.]

Reviewers' comments:

Reviewer's Responses to Questions

Comments to the Author

1. If the authors have adequately addressed your comments raised in a previous round of review and you feel that this manuscript is now acceptable for publication, you may indicate that here to bypass the “Comments to the Author” section, enter your conflict of interest statement in the “Confidential to Editor” section, and submit your "Accept" recommendation.

Reviewer #2: All comments have been addressed

Reviewer #3: All comments have been addressed

________________________________________

2. Is the manuscript technically sound, and do the data support the conclusions?

The manuscript must describe a technically sound piece of scientific research with data that supports the conclusions. Experiments must have been conducted rigorously, with appropriate controls, replication, and sample sizes. The conclusions must be drawn appropriately based on the data presented.

Reviewer #2: Yes

Reviewer #3: Yes

________________________________________

3. Has the statistical analysis been performed appropriately and rigorously?

Reviewer #2: Yes

Reviewer #3: Yes

________________________________________

4. Have the authors made all data underlying the findings in their manuscript fully available?

The PLOS Data policy requires authors to make all data underlying the findings described in their manuscript fully available without restriction, with rare exception (please refer to the Data Availability Statement in the manuscript PDF file). The data should be provided as part of the manuscript or its supporting information, or deposited to a public repository. For example, in addition to summary statistics, the data points behind means, medians and variance measures should be available. If there are restrictions on publicly sharing data—e.g. participant privacy or use of data from a third party—those must be specified.

Reviewer #2: Yes

Reviewer #3: Yes

________________________________________

5. Is the manuscript presented in an intelligible fashion and written in standard English?

PLOS ONE does not copyedit accepted manuscripts, so the language in submitted articles must be clear, correct, and unambiguous. Any typographical or grammatical errors should be corrected at revision, so please note any specific errors here.

Reviewer #2: Yes

Reviewer #3: Yes

________________________________________

6. Review Comments to the Author

Please use the space provided to explain your answers to the questions above. You may also include additional comments for the author, including concerns about dual publication, research ethics, or publication ethics. (Please upload your review as an attachment if it exceeds 20,000 characters)

Reviewer #2: The authors have strengthened the different sections of the paper. I have a few minor suggestions for consideration.

Authors’ overall response

The authors would like to thank the reviewer for the time and effort the reviewer invested to review this paper. We found all the suggestions are likely to improve our manuscript, therefore we have revised the manuscript as per suggestion of the reviewer.

- At the outset, it ay be good to state how common is internal migration in Bangladesh for the women.

Authors’ response

We have added the statistics at the relevant place. Please see the lines (70–72) in the file named Revised Manuscript with Track Changes.

- Line 97- Please state the directionality of the accessibility and equity. Was it positive?

Authors’ response

We have added the explanation about the directionality. Please see the lines 97–103 in the file Revised Manuscript with Track Changes.

- Line 111-118 - please remove the example. You could just state that international migration is very uncommon and therefore not considered in this paper.

Authors’ response

We have revised the lines as per suggestion. Please see the lines 116–126 in the file Revised Manuscript with Track Changes.

- Please change the wording of Line 444. I think we would want to learn from the urban health care settings and strengthen the rural health care structure to improve ANC and other services

Authors’ response

We have revised the line as per suggestion. Please see the lines 452–458 in the file Revised Manuscript with Track Changes.

Reviewer #3: The topic of this article is relevant and important. Authors have already addressed all reviewer comments

Authors’ response

Thank you very much for your time and consideration.

________________________________________

7. PLOS authors have the option to publish the peer review history of their article (what does this mean?). If published, this will include your full peer review and any attached files.

If you choose “no”, your identity will remain anonymous but your review may still be made public.

Do you want your identity to be public for this peer review? For information about this choice, including consent withdrawal, please see our Privacy Policy.

Reviewer #2: No

Reviewer #3: No

Attachment Submitted filename: Response_to_Reviewers_auresp_3.docx

# Editor report: Decision Letter 3

**DOI:** 10.1371/journal.pone.0345817.r007

**Authors:** Henk Schallig

11 Jun 2026

Influence of internal migration on antenatal care utilization in Bangladesh: Findings from a nationally representative cross-sectional survey

PONE-D-25-39205R3

Dear Dr. Rahman,

We’re pleased to inform you that your manuscript has been judged scientifically suitable for publication and will be formally accepted for publication once it meets all outstanding technical requirements.

Within one week, you’ll receive an e-mail detailing the required amendments. When these have been addressed, you’ll receive a formal acceptance letter and your manuscript will be scheduled for publication.

An invoice will be generated when your article is formally accepted. Please note, if your institution has a publishing partnership with PLOS and your article meets the relevant criteria, all or part of your publication costs will be covered. Please make sure your user information is up-to-date by logging into Editorial Manager at Editorial Manager® and clicking the ‘Update My Information' link at the top of the page. For questions related to billing, please contact billing support.

If your institution or institutions have a press office, please notify them about your upcoming paper to help maximize its impact. If they’ll be preparing press materials, please inform our press team as soon as possible -- no later than 48 hours after receiving the formal acceptance. Your manuscript will remain under strict press embargo until 2 pm Eastern Time on the date of publication. For more information, please contact onepress@plos.org.

Kind regards,

Henk Schallig, Ph.D

Academic Editor

PLOS One

Additional Editor Comments (optional):

Reviewers' comments:

# Editor report: Acceptance letter

**DOI:** 10.1371/journal.pone.0345817.r008

**Authors:** Henk Schallig

PONE-D-25-39205R3

PLOS One

Dear Dr. Rahman,

I'm pleased to inform you that your manuscript has been deemed suitable for publication in PLOS One. Congratulations! Your manuscript is now being handed over to our production team.

At this stage, our production department will prepare your paper for publication. This includes ensuring the following:

* All references, tables, and figures are properly cited

* All relevant supporting information is included in the manuscript submission,

* There are no issues that prevent the paper from being properly typeset

You will receive further instructions from the production team, including instructions on how to review your proof when it is ready. Please keep in mind that we are working through a large volume of accepted articles, so please give us a few days to review your paper and let you know the next and final steps.

Lastly, if your institution or institutions have a press office, please let them know about your upcoming paper now to help maximize its impact. If they'll be preparing press materials, please inform our press team within the next 48 hours. Your manuscript will remain under strict press embargo until 2 pm Eastern Time on the date of publication. For more information, please contact onepress@plos.org.

You will receive an invoice from PLOS for your publication fee after your manuscript has reached the completed accept phase. If you receive an email requesting payment before acceptance or for any other service, this may be a phishing scheme. Learn how to identify phishing emails and protect your accounts at https://explore.plos.org/phishing.

If we can help with anything else, please email us at customercare@plos.org.

Thank you for submitting your work to PLOS ONE and supporting open access.

Kind regards,

PLOS ONE Editorial Office Staff

on behalf of

Dr. Henk Schallig

Academic Editor

PLOS One
